# Comparative transcriptomics and proteomics analysis of the symbiotic germination of *Paphiopedilum barbigerum* with *Epulorhiza* sp. FQXY019

**DOI:** 10.3389/fmicb.2024.1358137

**Published:** 2024-03-18

**Authors:** Fan Tian, Juncai Wang, Fangjun Ding, Lianhui Wang, Yanbing Yang, Xinxiang Bai, Chengjiang Tan, Xiaofeng Liao

**Affiliations:** ^1^Guizhou Academy of Forestry, Guiyang, Guizhou, China; ^2^Key Laboratory for Biodiversity Conservation in the Karst Mountain Area of Southwestern China, National Forestry and Grassland Administration, Guiyang, Guizhou, China; ^3^Guizhou Academy of Sciences, Guiyang, Guizhou, China; ^4^College of Forestry, Guizhou University, Guiyang, Guizhou, China; ^5^Guizhou Maolan National Nature Reserve Administration, Libo, Guizhou, China

**Keywords:** *Paphiopedilum barbigerum*, mycorrhizal fungi, symbiotic germination, transcriptome, proteome

## Abstract

**Introduction:**

*Paphiopedilum barbigerum* is currently the rarest and most endangered species of orchids in China and has significant ornamental value. The mature seeds of *P. barbigerum* are difficult to germinate owing to the absence of an endosperm and are highly dependent on mycorrhizal fungi for germination and subsequent development. However, little is known about the regulation mechanisms of symbiosis and symbiotic germination of *P. barbigerum* seeds.

**Methods:**

Herein, transcriptomics and proteomics were used to explore the changes in the *P. barbigerum* seeds after inoculation with (FQXY019 treatment group) or without (control group) *Epulorhiza* sp. FQXY019 at 90 days after germination.

**Results:**

Transcriptome sequencing revealed that a total of 10,961 differentially expressed genes (DEGs; 2,599 upregulated and 8,402 downregulated) were identified in the control and FQXY019 treatment groups. These DEGs were mainly involved in carbohydrate, fatty acid, and amino acid metabolism. Furthermore, the expression levels of candidate DEGs related to nodulin, Ca^2+^ signaling, and plant lectins were significantly affected in *P. barbigerum* in the FQXY019 treatment groups. Subsequently, tandem mass tag-based quantitative proteomics was performed to recognize the differentially expressed proteins (DEPs), and a total of 537 DEPs (220 upregulated and 317 downregulated) were identified that were enriched in processes including photosynthesis, photosynthesis-antenna proteins, and fatty acid biosynthesis and metabolism.

**Discussion:**

This study provides novel insight on the mechanisms underlying the *in vitro* seed germination and protocorm development of *P. barbigerum* by using a compatible fungal symbiont and will benefit the reintroduction and mycorrhizal symbiotic germination of endangered orchids.

## 1 Introduction

The *Orchidaceae* is one of the largest and most diverse families of flowering plants in the world, with >28,000 accepted species across 800 genera (Chao et al., 2014; Zhao et al., 2021). Orchids are considerably valuable from ecological, ornamental, medical, and evolutionary perspectives (Zhang et al., 2018; Yang et al., 2020). *Paphiopedilum* is one of the most primordial genera of Orchidaceae, which includes 107 species so far. Members of this genus have considerable horticultural value and development potential (Huang et al., 2016; Fang et al., 2021). The *Paphiopedilum* orchids are generally known as the Lady’s slipper orchid owing to their slipper-shaped pouch (Tsai et al., 2020). They are extremely popular and highly valued in the horticulture market because of their unique flowers that have abundant colors, shapes, and sizes and long floral lifespan (Tsai et al., 2020; Fang et al., 2021). Unfortunately, members of this genus are endangered with their population severely declining in recent decades owing to the native habitat loss, rampant smuggling, and over-collection (Fang et al., 2021). To conserve these endangered species, the Convention on International Trade in Endangered Species of Wild Fauna and Flora (CITES) placed all *Paphiopedilum* species on the conservation list in 2017 to ban illegal trade (Yang et al., 2020). Furthermore, the mature *Paphiopedilum* seeds are very small, have few nutrient reserves, and lack an endosperm and are consequently difficult to germinate under natural conditions, making them particularly vulnerable to extinction (Zhang et al., 2018; Mujica et al., 2021). Therefore, the *Paphiopedilum* seed germination requires investigation, which is important for appropriately utilizing and conserving species resources.

The traditional propagation of *Paphiopedilum* orchids using an axillary bud division from the mother plant is unproductive and time-consuming (Chen et al., 2004). Many *Paphiopedilum* seeds form a compatible and symbiotic relationship with mycorrhizal or endophytic fungi that facilitates their germination in conditions of their natural habitat (Xu et al., 2020; Li Y. Y. et al., 2021). During this process, regarded as symbiotic germination, fungi and seeds form a nonphotosynthetic spherical body named a protocorm, which completely relies on mycorrhizal fungi for nourishment (Fang et al., 2021). Therefore, symbiotic germination can be used as an ordinary and efficient approach for promoting the germination and the large-scale propagation of *Paphiopedilum* (Chen et al., 2004). Several studies have found that mycorrhizal fungi can provide carbon and nitrogen sources, amino acids, and other nutrients as well as metabolic regulatory substances such as hormones for the seed germination of *Paphiopedilum* plants, whereas the plants provide carbohydrates for the mycorrhizal fungi (Fochi et al., 2017; Mujica et al., 2021; Zhao et al., 2021). Furthermore, the existence of compatible mycorrhizal fungi in *Paphiopedilum* seedlings enhances the adaptability of plants to the environment to increase plant survival and growth rate (Zhang et al., 2018). However, *Paphiopedilum* seedlings do not simply form a symbiotic relationship with any fungus, and germination can only happen when an appropriate fungus is present (Pujasatria et al., 2020), which includes *Tulasnellaceae*, *Ceratobasidiaceae*, *Serendipitaceae*, *Mycena*, *Helicogloea*, *Fusarium*, or other fungi (Xu and Guo, 1989; Kottke et al., 2010; Sebastián et al., 2014; Jiang et al., 2019). Understanding the interaction between *Paphiopedilum* and mycorrhizal fungi is a significant for generative reproduction, augmenting existing populations, and maintaining species diversity in orchids (Yang et al., 2020). Currently, research on the interaction between mycorrhiza and *Paphiopedilum* plants mainly includes promoting seed germination, growth and development, nutrient absorption, improving stress resistance of plants and the symbiotic relationship (Gao et al., 2022; Tian et al., 2023; Xu et al., 2023; Yang H. et al., 2023; Yang J. X. et al., 2023), whereas the molecular regulation mechanism of the interaction underlying the symbiotic relationship need further investigation.

In recent decades, understanding of the symbiotic interaction mechanism between mycorrhizal fungi and orchids has entered a new era with the development of next-generation sequencing and multi-omics technologies (Chen et al., 2018, 2022; Ahmad et al., 2022a). Multi-omics technology (i.e., transcriptomics, proteomics, and metabolomics) has become an effective method of exploring the orchid-fungus relationship (Balilashaki et al., 2019). For example, Fochi et al. (2017) used transcriptomics analysis to identify two functional ammonium transporters and several amino acid transporters involved in nitrogen uptake and transfer to host plants by the orchid mycorrhiza fungus *Tulasnella calospora.* Ghirardo et al. (2020) applied untargeted metabolomics to report that many metabolites, especially lipid compounds, were significantly altered during the symbiotic relationship between the Mediterranean orchid *Serapias vomeracea* and the basidiomycete *T. calospora*. In addition, Chen et al. (2018) used proteomic analysis to identify 42 proteins associated with antioxidant pathways, protein metabolism, and energy metabolism in *Phalaenopsis*. Hence, the multi-omics techniques are regarded as a promising approach for exploring the molecular mechanisms of the symbiotic relationship between mycorrhizal fungi and orchids.

Herein, we investigated the germination of *P. barbigerum,* a rare *Paphiopedilum* native to southwest China with high ornamental value, in the presence of a mycorrhizal fungus, *Epulorhiza* sp. FQXY019, which we had previously isolated and which progressed seed germination of *P. barbigerum* (Tian et al., 2022). Based on this, we integrated transcriptomics analyses with proteomic techniques to investigate the genes and proteins differentially expressed in the seed germination of *P. barbigerum* with and without Epulorhiza sp. FQXY019 inoculation. Thus, this study aimed to (1) reveal the transcriptional changes in the major metabolic pathways, and (2) investigate the differences in the proteomic profiles of *P. barbigerum* inoculated and uninoculated *Epulorhiza* sp. FQXY019. We hypothesized that: FQXY019 would induce the different biological and metabolic pathways of the *P. barbigerum* seed germination, and activate numerous responsive genes and proteins involved in molecular pathways at transcriptomic and proteomic level. The results will provide a theoretical basis for further elucidating the molecular regulation mechanism of the symbiotic relationship between orchids and mycorrhizal fungi as well as for the propagation and conservation of orchids.

## 2 Materials and methods

### 2.1 *Paphiopedilum barbigerum* seed collection and growth of the free-living mycelium

In January 2021, the mature and well-grown fruit capsules were collected from *P. barbigerum* plants in the greenhouse of the Institute of Biotechnology, Guizhou Academy of Forestry. Capsules were desiccated at room temperature and stored in a glass vessel at 4°C until sowing. *Epulorhiza* sp. FQXY019 had been isolated from the mycorrhizal roots of *P. barbigerum* in our previous study (Tian et al., 2022). First, a layer of cellophane was placed onto 9 cm Petri dishes containing 30 mL potato dextrose agar (PDA) medium (200 g potato, 10 g agar, 20 g glucose, and 1 L distilled water). *Epulorhiza* sp. FQXY019 was then inoculated onto the cellophane. After 2 weeks of culture, the healthy mycelium of the fungus filled the Petri dishes and was used for the symbiotic seed germination assay.

### 2.2 Symbiotic and asymbiotic germination of *Paphiopedilum barbigerum* seeds

Capsules of *P. barbigerum* were surface sterilized in 70% ethanol for 30 s, and shook for 6 min in 0.1% mercuric chloride, before being washed five times with sterile distilled water (Tian et al., 2022). Subsequently, the fruit capsules were cut with a sterile scalpel and forceps, gently shaken, and approximately 100 seeds were evenly sown on *Epulorhiza* sp. FQXY019 mycelium for symbiotic germination testing as the FQXY019-treatment group. Symbiotic seed germination was obtained by placing surface-sterilized seeds directly on PDA culture medium without *Epulorhiza* sp. FQXY019 as the control group (CK). Each treatment had 30 plates, which represented three replicates. The average temperature of culture was 25/20°C (darkness/light), light intensity was 1,200 lux, and day/night photoperiod was 18/6 h (Meng et al., 2020; Tian et al., 2022). Symbiotic and asymbiotic plants were collected after culturing for 90 days. At this point, the seeds had germinated, and the seedlings had two or three leaves. Sampling (the whole seedling) was performed after 90 days of culture because we had previously found that the seeds of *P. barbigerum* inoculated with *Epulorhiza* sp. FQXY019 can further develop, and rooting occurred after 90 days of culture (Tian et al., 2022). The figures of plant growing with and without *Epulorhiza* sp. FQXY019 was showed in Supplementary Figure S1. Three biological replicates were collected for each experimental group. All samples were quickly flash-frozen in liquid nitrogen and stored at −80°C for further analysis.

### 2.3 RNA extraction, sequencing, and *de novo* assembly

Total RNA was extracted from approximately 200 mg of frozen *P. barbigerum* plant samples of each treatment using prechilled RNA extraction Kit (TIANGEN, No DP411, in China) following the manufacture’s protocols (Ahmad et al., 2022a). The extracted RNA concentration and purity were assessed using a spectrophotometer (NanoDrop 2000, Thermo Fisher Scientific), and the integrity of RNA was assessed using an Agilent Bioanalyzer 2,100 system (Agilent Technologies, USA) (Mumtaz et al., 2022). Three separate libraries were prepared from three biological replicates per treatment. An RNA integrity number > 8.0 was selected to construct the sequencing library. cDNA libraries were constructed using the TruSeq™ RNA sample prep kit (Illumina, San Diego, USA) following the manufacturing specification after mRNA was isolated from total RNA using oligo (dT) magnetic beads and broken randomly into numerous fragments. Subsequently, sequencing was performed using an NovaSeq 6,000 platform by Beijing Biomarker Technologies Co., Ltd. To obtain high-quality clean reads, the remaining adapters, ploy-N, and low-quality value raw reads were eliminated (Ahmad et al., 2022a). High-quality clean reads were further *de novo* assembled using Trinity software. The unigenes were annotated in accordance with the NR, GO, KEGG, KOG, Pfam, SwissProt, TrEMBL, and eggNOG databases. The raw data of transcriptomics were available in the NCBI under accession PRJNA868469.

#### 2.3.1 Differential expression genes and enrichment analysis

Gene expression levels were assessed using the fragments per kilobase per transcript per million mapped reads (FPKM). To consider the correlation of gene expression levels between control and treatment group samples, Pearson’s correlation coefficient was evaluated. Screening standards for differentially expressed genes (DEGs) were |log2(fold change, FC)| > 1 and false discovery rates (FDR) < 0.01 (Li P. et al., 2021). DEGs were identified utilizing the DEseq R package. To deduce the supposed function of DEGs, we performed GO enrichment analysis and KEGG pathway analysis using the topGO R package and KOBAS software, respectively.

#### 2.3.2 qRT-PCR validation

To confirmation the accuracy of RNA-seq data, 12 DEGs were randomly chosen for qRT-PCR validation. Primers were designed via Primer Premier version 5.0 and are listed in Supplementary Table S1. qRT-PCR was performed as reported by Li P. et al. (2021). The calculation method of 2^−ΔΔCt^ was employed for the relative expression level of genes. Three biological replicates were measured per gene, and actin was used as a reference gene.

### 2.4 Proteomic extraction and tandem mass tag quantification

Plant material protein isolation and Tandem Mass Tag (TMT) label-based proteome analyzes were conducted by Beijing Biomarker Technologies Co., Ltd. The proteins of *P. barbigerum* were extracted as described by Hao et al. (2017) and Zhu et al. (2021). Briefly, frozen samples were ground into a fine powder under liquid nitrogen. Then, the powder was transferred into a centrifuge tube and sonicated on ice three times in lysis buffer of 8 M urea, 2 mM EDTA, 10 mM dithiothreitol (DTT), and 1% protease inhibitor cocktail. The sediment was pelleted by centrifugation at 12,000 × *g* for 20 min. The protein in the supernatant was deposited with 15% precooled TCA buffer for 2 h at −20°C. The supernatant was removed, and then residual sediment was rinsed with cold acetone. Finally, the protein was redissolved in 8 M urea/100 mM tetraethylammonium bromide buffer (pH 8.0). The amount of protein in each sample was measured via the Bradford method and examined by sodium dodecyl sulfate polyacrylamide gel electrophoresis.

The protein sample was reduced with 10 mM DTT for 1 h at 37°C followed by an alkylation reaction at room temperature for 45 min by incubation in 40 mM iodoacetamide without light. Subsequently, the protein solution was diluted with 100 mM TEAB to a urea concentration of <2 M. Trypsin was added at a ratio of 1:50 (enzyme: protein, w/w) for the first digestion overnight and then was secondly digested for 4 h at a ratio of 1:100. After digestion, the protein samples were peptide desalted using a Strata X C18 SPE column and vacuum dried. The peptide was reconstituted with 0.5 M TEAB in accordance with the manufacturer’s protocol for the TMT kit (Thermo-Scientific, United States). Briefly, one unit of TMT reagent was reconstituted in acetonitrile. The peptide mixtures were incubated at room temperature for 2 h, and merged, desalted, and dried by vacuum centrifugation (Grosjean et al., 2022).

#### 2.4.1 HPLC fractionation and LC–MS/MS analysis

The sample was fractionated and purified using high-pH reverse-phase HPLC via an Agilent Zorbax 300Extend-C18 column (250 × 4.6 mm, 5-mm particles). Peptides were divided into eighty fractions over 80 min using a 2–60% acetonitrile gradient in 10 mM ammonium bicarbonate (pH 10). Peptides were collected into 18 fractions and dried via vacuum centrifugation (Jian et al., 2020).

Peptide samples were redissolved in 0.1% formic acid (FA, buffer A) and then loaded onto an EASY-nLC 1,000 UPLC system (Thermo-Scientific, USA) equipped with an Agilent Zorbax 300Extend-C18 column as above. Peptides were eluted with a linear gradient from 7 to 26% solvent B (1% FA in 98% acetonitrile) for 22 min, 26 to 40% in 12 min and 40 to 80% in 3 min, then remaining at 80% for the last 3 min, at a flow rate of 400 nL/min. Target peptides were analyzed using mass spectrometry (MS/MS) with a Q Exactive™ Plus (Thermo-Scientific, USA) with a Nanospray Flex™ (NSI) source. The electrospray voltage applied was 2.4 kV. Full-scan MS spectra (m/z 400–1,500) were examined in an Orbitrap at a mass resolution of 60,000 at m/z 200. A data-dependent procedure was conducted with 15-s dynamic exclusion of one MS scan followed by 20 MS/MS scans. Automatic gain control (AGC) was set as 3e6. After the full scan, the top 40 intense precursor ions were chosen for MS2 analysis at a resolution of 17,500 at m/z 200. The MS2 activation type was higher-energy collisional dissociation, normalized collision energy was 30 eV; AGC was set as 5e4. The obtained MS/MS data were evaluated with the Maxquant search engine (v.1.5.2.8). Multiple *p* values were controlled to a FDR of 1%. Protein quantification analysis was conducted on at least two unique peptides, and protein confidence was set at a 95% confidence interval (Jian et al., 2020; Zhou et al., 2022).

#### 2.4.2 Bioinformatics analysis

DEPs were identified using the thresholds of |log2(fold change, FC)| > 1 and *p*-value <0.05 in three biological replicates. The relative expression of the studied proteins was utilized to perform hierarchical clustering analysis by Cluster 3.0. GO functional annotation of the proteome was derived from the UniProt-GOA database using the Blast2GO program. DEPs were divided into three categories: BP, CC, and MF based on GO terms. The KEGG database was used to categorize these screened protein species via Blastx/Blastp 2.2.24 software. Then, the pathway enrichment of GO and KEGG of the DEPs was conducted using the Fisher’ exact test, and pathways with a p-value <0.05 were considered significant (Peng et al., 2019; Yu et al., 2019).

## 3 Results

### 3.1 Transcriptome assembly

Three independent replicates of *P. barbigerum* in the control (CK) and FQXY019 treatment groups were analyzed via RNA-seq to reveal the potential symbiosis mechanism. The number of clean reads generated from a total of six libraries (three repeats per treatment) ranged from 22,375,505 to 24,054,439, with a GC content >47.86%; the percentage of Q20, Q30. and mapped ratio value was more than 97.77, 93.77, and 73%, respectively (Supplementary Table S2). After assembling the high-quality reads, the total number of transcripts and unigenes were 175,386 and 100,136 with a mean length of 1071.96 and 800.22 bp and an N50 length of 1842 and 1,577 bp, respectively (Supplementary Table S3). Overall, these results illustrated the high quality of the RNA-Seq data and practicability for further analysis.

All 40,742 unigenes were annotated via eight public databases, and 39,850 (97.81%), 30,367 (74.53%), 23,334 (57.27%), 20,217 (49.62%), 24,422 (59.94%), 20,781 (51.01%), 36,768 (90.25%), and 30,161 (74.03%) unigenes were successfully annotated to known proteins in the National Centre for Biotechnology Information (NCBI) non-redundant protein sequences (NR), Gene Ontology (GO), Kyoto Encyclopedia of Genes and Genomes (KEGG), eukaryotic Orthologous Groups (KOG), Protein family (Pfam), Swiss-prot protein Sequence (Swiss-Prot), TrEMBL, and Evolutionary Genealogy of Genes: Nonsupervised Orthologous Groups (eggNOG) databases, respectively (Supplementary Table S4). The homologous species distribution analysis showed that 29.49% of annotated unigenes were matched with *Dendrobium catenatum*, in the *Orchidaceae* family (Supplementary Figure S2). This result implied that some gene sequence results of *D. catenatum* could be used as reference in the further study of the molecular mechanism of the symbiotic germination of *P. barbigerum* seeds as *P. barbigerum* is a reference-free genome plant. In addition, since these two plants belong to different genera, many unknown functional genes may be present in *P. barbigerum* that are different from those in *D. catenatum* and need further exploration.

**Figure 1:**
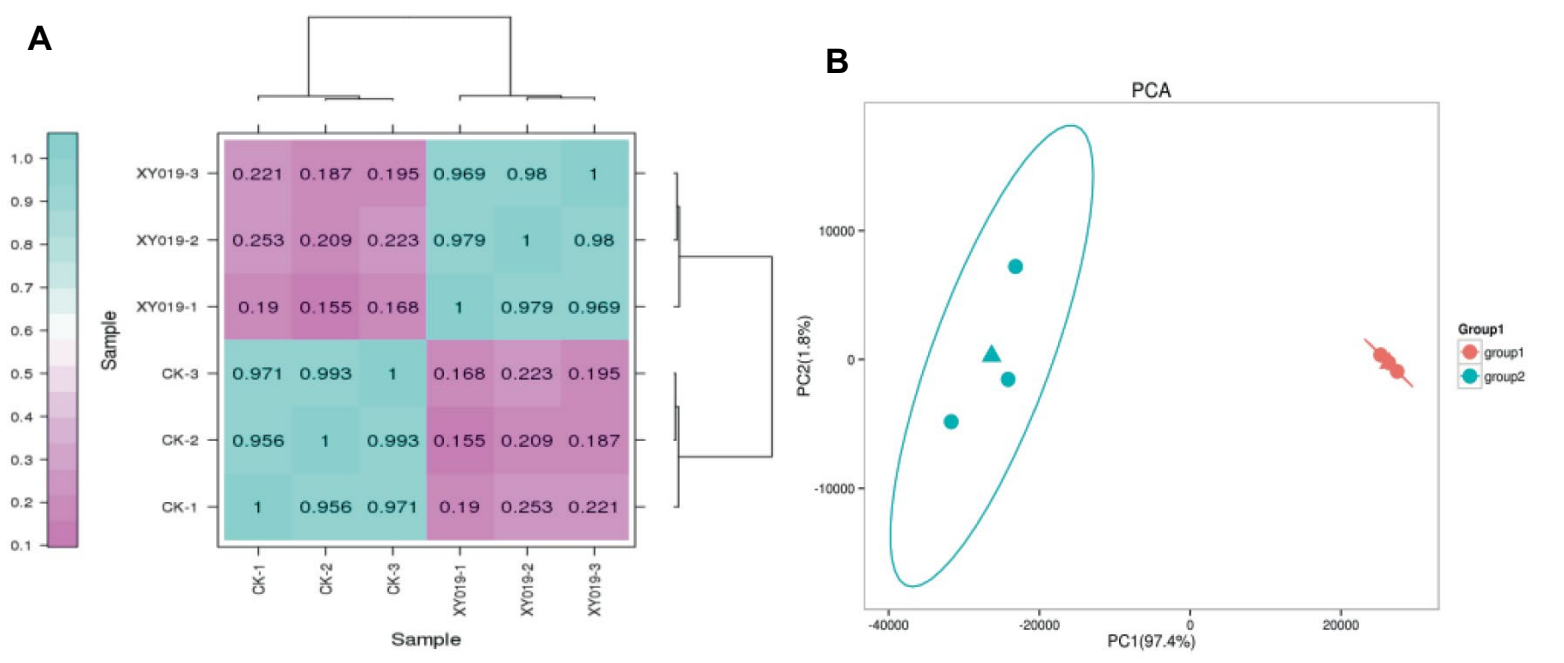
**(A)** Correlation analysis of the gene expression patterns in the FQXY019 treatment and CK groups. **(B)** Principal component analysis (PCA) of FPKM profiles in the FQXY019 treatment groups (group 1) and CK (group 2). The deeper the blue represents the stronger the positive correlation, and the deeper the purple the weaker the positive correlation **(A)**. The blue and orange triangles represent the average coordinates of the three groups **(B)**.

A correlation coefficient (R^2^) of biological samples between 0.8 and 1 is a strong correlation relationship (*p* < 0.05), and when R^2^ is <0.8, the correlation relationship between samples is low. In the case of the gene expression patterns in each treatment, the R^2^ among the three replicates was >0.9, implying a remarkably high correlation (Figure 1A). Meanwhile, principal component analysis (PCA) suggested that the expression of gene clusters in the control and inoculation treatments was distinguishable, indicating a distinct responsive effect of *Epulorhiza* sp. FQXY019 inoculation on the gene expression patterns (Figure 1B).

### 3.2 Identification and enrichment analysis of DEGs

**Figure 2:**
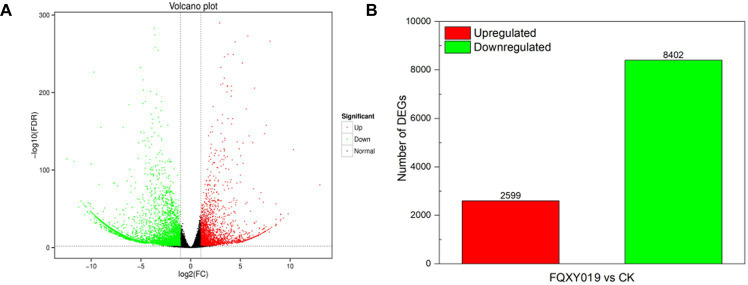
Differentially expressed genes (DEGs) for FQXY019 treatment groups vs. control (CK). **(A)** Volcano plot and **(B)** histogram; the number of up- and downregulated genes are represented by red and green, respectively.

**Figure 3:**
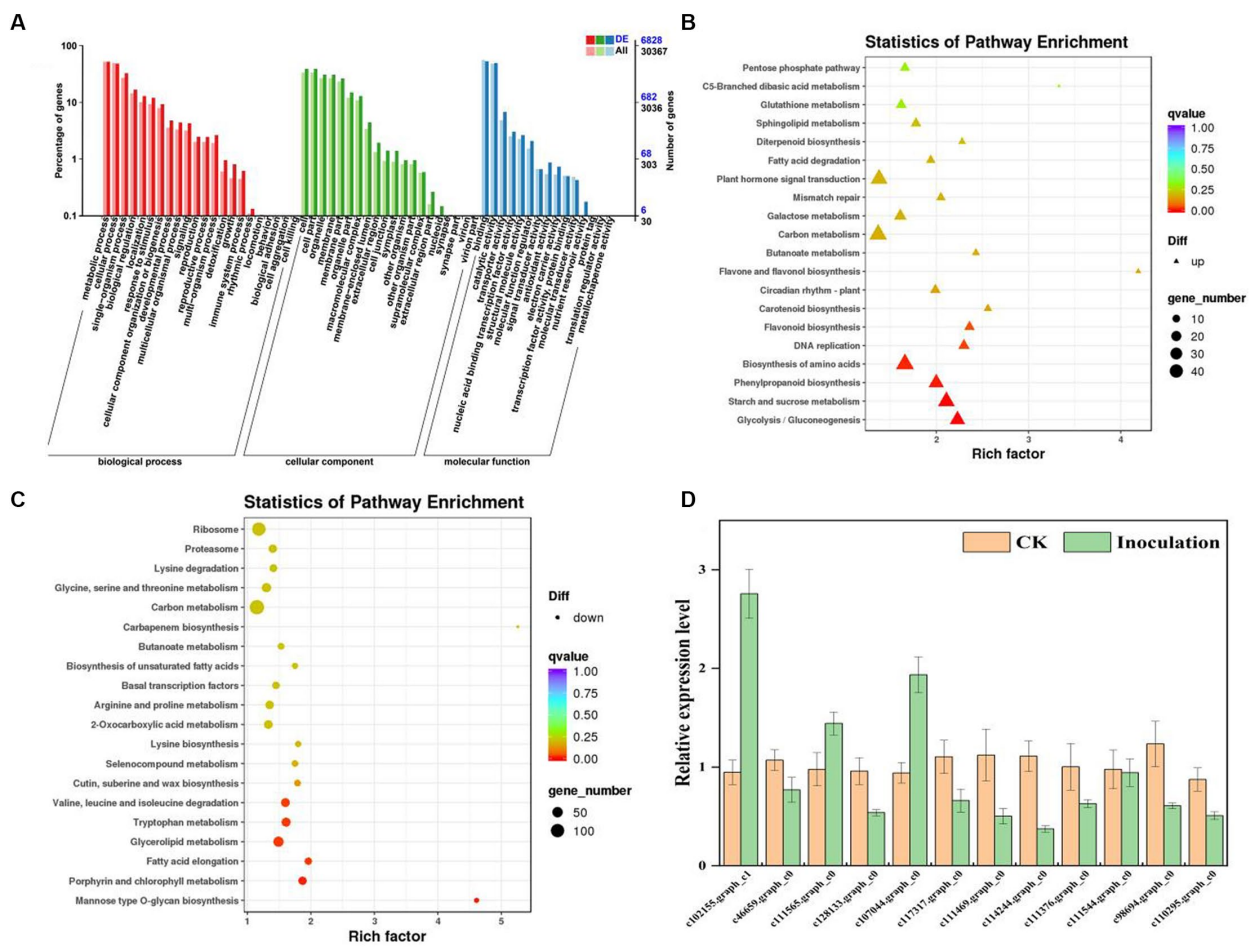
Graphical representation of significantly enriched pathways. **(A)** GO enrichment analysis of DEGs for FQXY019 treatment groups vs. CK. The bubble diagram shows the top 20 significant enriched pathways in the upregulated **(B)** and downregulated **(C)** DEGs for FQXY019 treatment groups vs. CK obtained via KEGG analysis. **(D)** qRT-PCR validation of DEG results. Values of qRT-PCR are mean ± SD (n = 3). Bars indicate SD. The detailed primer information is shown in Supplementary Table S1. The X-axis was the rich factor, which was the ratio of the DEG number to the total gene number in a certain pathway; the Y-axis represents the name of the pathway. The bubble size represents the number of DEGs involved. The bubble color indicates the enrichment degree of the pathway.

To identify DEGs in the two groups, we used a pairwise comparison. As shown in Figure 2, a total of 10,961 DEGs (2,599 upregulated and 8,402 downregulated) was identified in CK compared with expression in the FQXY019-treatment group (Supplementary Table S5). Subsequently, we annotated these DEGs using the GO database to investigate their functions. According to GO enrichment, a total of 6,828 DEGs were enriched to 57 GO terms, including 22, 20, and 15 terms in biological process (BP), cellular component (CC), and molecular function (MF), respectively (Figure 3A). Among them, the metabolic, cellular, and single-organism processes were the top three subcategories in BP; cell, cell part, and organelle were the top three subcategories in CC; and binding, catalytic activity, and transporter activity were the top three subcategories in MF (Figure 3A). Therefore, the DEGs between the two treatment groups were mainly involved in these processes, revealing that inoculation had an effect on these pathways.

To reveal the biological functions and associated pathways of DEGs in *Epulorhiza* sp. FQXY019-induced symbiotic relationships, KEGG enrichment analysis was performed. Most DEGs were mainly annotated in metabolic pathways (Figures 3A–C; Supplementary Figure S3 and Supplementary Table S6), indicating that these pathways are important for symbiotic germination of *P. barbigerum* seeds. The top three most significant KEGG pathways were glycolysis/gluconeogenesis, starch and sucrose metabolism, and phenylpropanoid biosynthesis in upregulated DEGs in CK, compared with the FQXY019 treatment group (Figure 3B; Supplementary Figures S4–S6). The DEGs involved in glycolysis/gluconeogenesis, such as *PFK, PFP, PDC, PGAM*, etc. were significantly upregulated, but the DEGs (*FBA, PDHA, DLD, FBP,* etc.) were downregulated in the FQXY019 treatment. The DEGs (*ISA, WAXY, E 2.4.1.14, etc*) related to starch and sucrose metabolism were significantly upregulated and the DEGs (*GYG1, SUS, INV, ENPP1, etc*) were downregulated in the FQXY019 treatment. The genes (*CCR, 4CL, CAD, COMT,* etc.) of phenylpropanoid biosynthesis were significantly upregulated, and the genes (*4CL, HCT, CCR,* etc.) were significantly downregulated in the FQXY019 treatment relative to CK treatment (Supplementary Figures S4–S6). By contrast, the mannose type O-glycan biosynthesis, porphyrin and chlorophyl II metabolism, and fatty acid elongation pathways were ranked the top three significantly enriched pathways in downregulated DEGs in CK compared with that in the FQXY019 treatment group (Figure 3C; Supplementary Figures S7–S9). In addition, the largest number of upregulated DEGs was in amino acid biosynthesis, carbon metabolism, and plant hormone signal transduction, whereas the largest number of downregulated DEGs was in carbon metabolism and ribosome and amino acid biosynthesis. This suggests that the inoculation of FQXY019 affected the growth of seedlings by regulating metabolic pathways, including those of carbon and amino acid metabolism. In summary, these results imply that carbohydrate, fatty acid, and amino acid metabolism are the main physiological activity pathways used to maintain the stability of symbiotic relationships during the germination of *P. barbigerum* seeds.

### 3.3 Validation of the DEG results via qRT-PCR analysis

To validate the reliability of the transcriptome results, we randomly selected 12 DEGs and examined them by qRT-PCR. According to the qRT-PCR analysis, the expression trends of 12 DEGs except in two DEGs (c46659.graph_c0 and c128133.graph_c0) were consistent with those obtained via transcriptome analysis (Figure 3D). The qRT-PCR results demonstrated that the transcriptome results were reliable and suitable for subsequent analysis.

### 3.4 Protein identification and comparison

**Figure 4:**
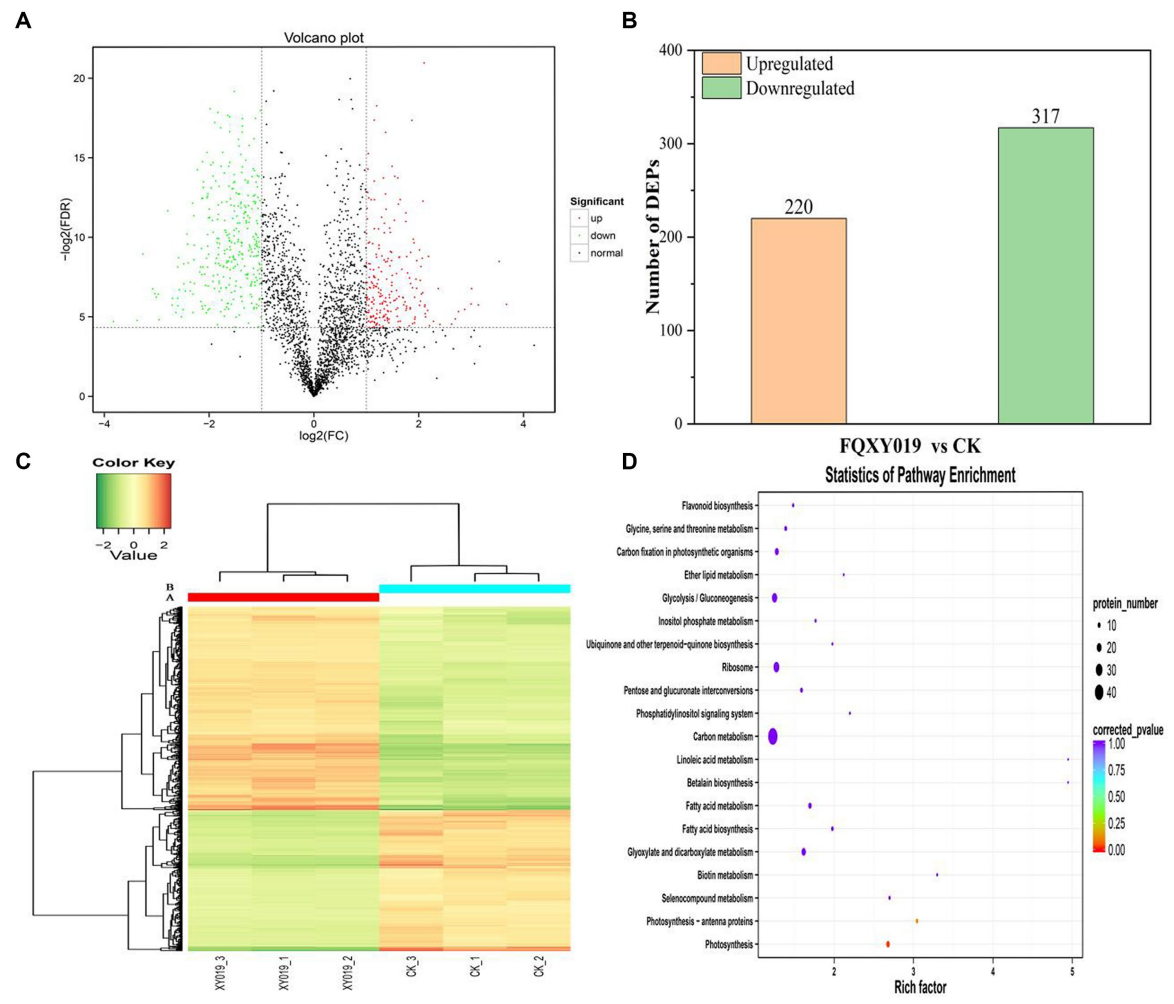
Differentially expressed proteins (DEPs) for FQXY019 treatment groups vs. control (CK). **(A)** Volcano plot and **(B)** histogram; the number of up- and downregulated genes are represented by red and green, respectively. **(C)** Hierarchical clustering analysis of the DEPs in different treatments. CK was the control treatment and FQXY019 was the inoculation treatment. Blue represents the CK, and red represents the FQXY019 treatment groups. **(D)** Bubble diagram showing the top 20 pathways with significant enrichment of DEPs for FQXY019 treatment groups and CK on KEGG pathway analysis. The X-axis was the rich factor, which was the ratio of the DEP number to the total protein number in a certain pathway; the Y-axis represents the name of the pathway. The bubble size represents the number of DEPs involved. The color of the circle represents *p*-value, which indicates the enrichment degree of the pathway.

To determine and identify up- or downregulated proteins in the *P. barbigerum* proteome inoculated with *Epulorhiza* sp. FQXY019 mycelium, we performed tandem mass tag (TMT) quantitative proteomic profiling of 90-day-old *P. barbigerum* seedlings in the CK and FQXY019 treatment groups, each with three biological replicates. After the combination of the two treatments of samples, 2,614 proteins were identified and quantified (Supplementary Table S10). Pairwise comparison of proteins with the criteria of a |log2(fold change, FC)| > 1 and a *p*-value <0.05 were screened as differentially expressed proteins (DEPs, Figure 4A). As shown in Figure 4B, 537 DEPs (220 upregulated and 317 downregulated) were detected in CK compared with the FQXY019 treatment group.

### 3.5 Hierarchical clustering and enrichment analysis of DEPs

We conducted a hierarchical clustering analysis of the DEPs in different treatments to explore the similarity between them. Each of the three replicates of the FQXY019 treatment groups and CK samples were clustered together independently and had high repeatability (Figure 4C). The protein abundance and the diversity of expression levels were markedly different between the FQXY019 treatment groups and the CK sample. PCA also showed similar results (Supplementary Figure S10).

### 3.6 Go and KEGG analysis of DEPs

To investigate the biological function of the DEPs, enrichment-based functional annotation was employed. GO functional annotation was performed on these DEPs, and 486 DEPs were successfully enriched into three categories including BP, CC, and MF. In the category of BP, the top three GO terms were metabolic process, cellular process, and single-organism process (Supplementary Figure S11a); the cell, cell part, and organelle were the largest subcategories in the CC category (Supplementary Figure S11b); and the top three GO terms were catalytic activity, binding, and structural molecule activity in MF analysis, respectively (Supplementary Figure S11c). Photosynthesis (BP), chloroplast thylakoid membrane (CC), and chlorophyl II binding (MF) were the most significantly enriched terms in these three processes (Supplementary Figure S11).

**Figure 5:**
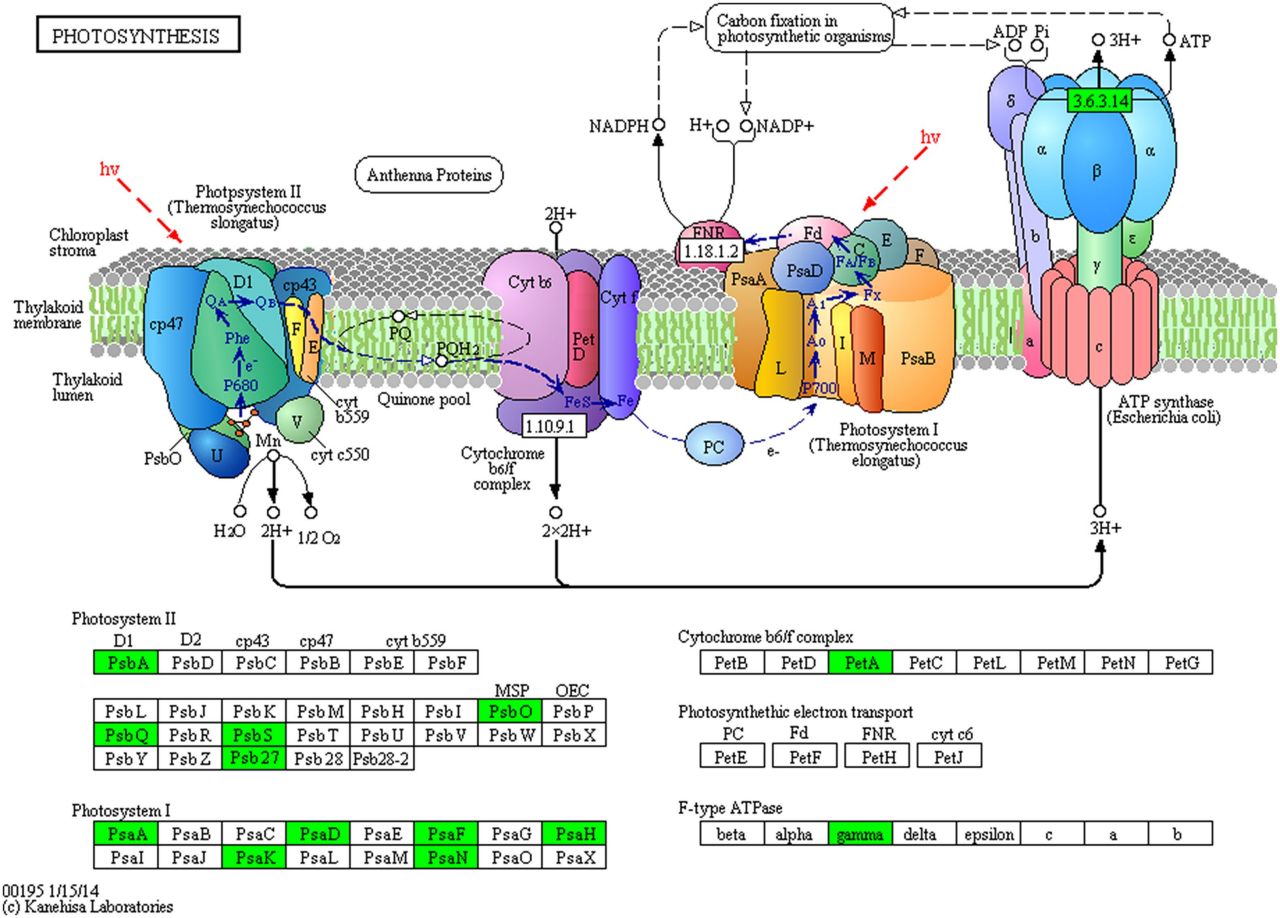
The expression levels of common DEPs in *P. barbigerum* involved in photosynthesis.

**Figure 6:**
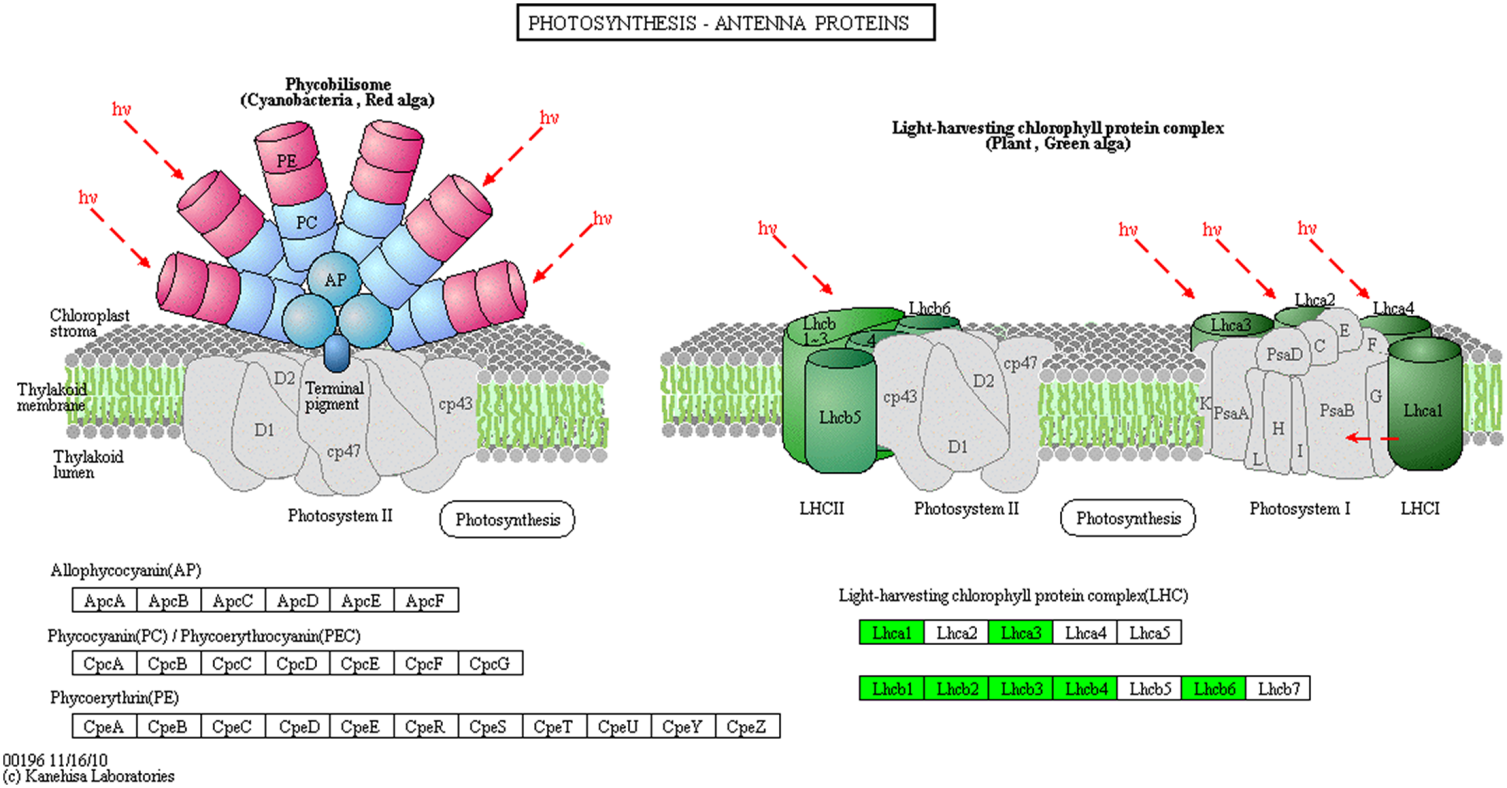
The expression levels of common DEPs in *P. barbigerum* related to photosynthesis-antenna proteins.

Proteins cannot accomplish their functions independently in an organism, and different proteins must coordinate with each other to achieve a series of biochemical reactions to conduct their biological functions. We therefore performed KEGG pathway enrichment analysis to help us understand how these DEPs were related to metabolism or signaling pathways and to reveal the biological functions and effect factors involved in the process. The DEPs were involved in 102 metabolic pathways, of which the top 20 pathways are shown in Figure 4D, including carbon metabolism, photosynthesis, amino acid biosynthesis, and fatty acid metabolism. Notably, the DEPs were primarily involved in metabolic pathways (Supplementary Figure S12); and the photosynthesis and photosynthesis-antenna proteins pathways were significantly enriched (Figures 4D, 5, 6). The DEPs (*PsaA, PsaD, PsaK, PsaF, etc*) related to photosynthesis I pathway and the DEPs (*PsbA, PsbO, PsbQ, Psb27, etc*) related to photosynthesis II pathway were significantly downregulated in the FQXY019 treatment compared with the CK treatment. The DEPs (*Lhca1, Lhca3, Lhcb1, Lhcb2,* etc.) involved in light-harvesting chlorophyl II protein complex pathway was significantly downregulated. These results indicating that symbiosis with *Epulorhiza* sp. FQXY019 could affect seed germination and seedling growth via changing the photosynthesis of *P. barbigerum.*

## 4 Discussion

**Figure 7:**
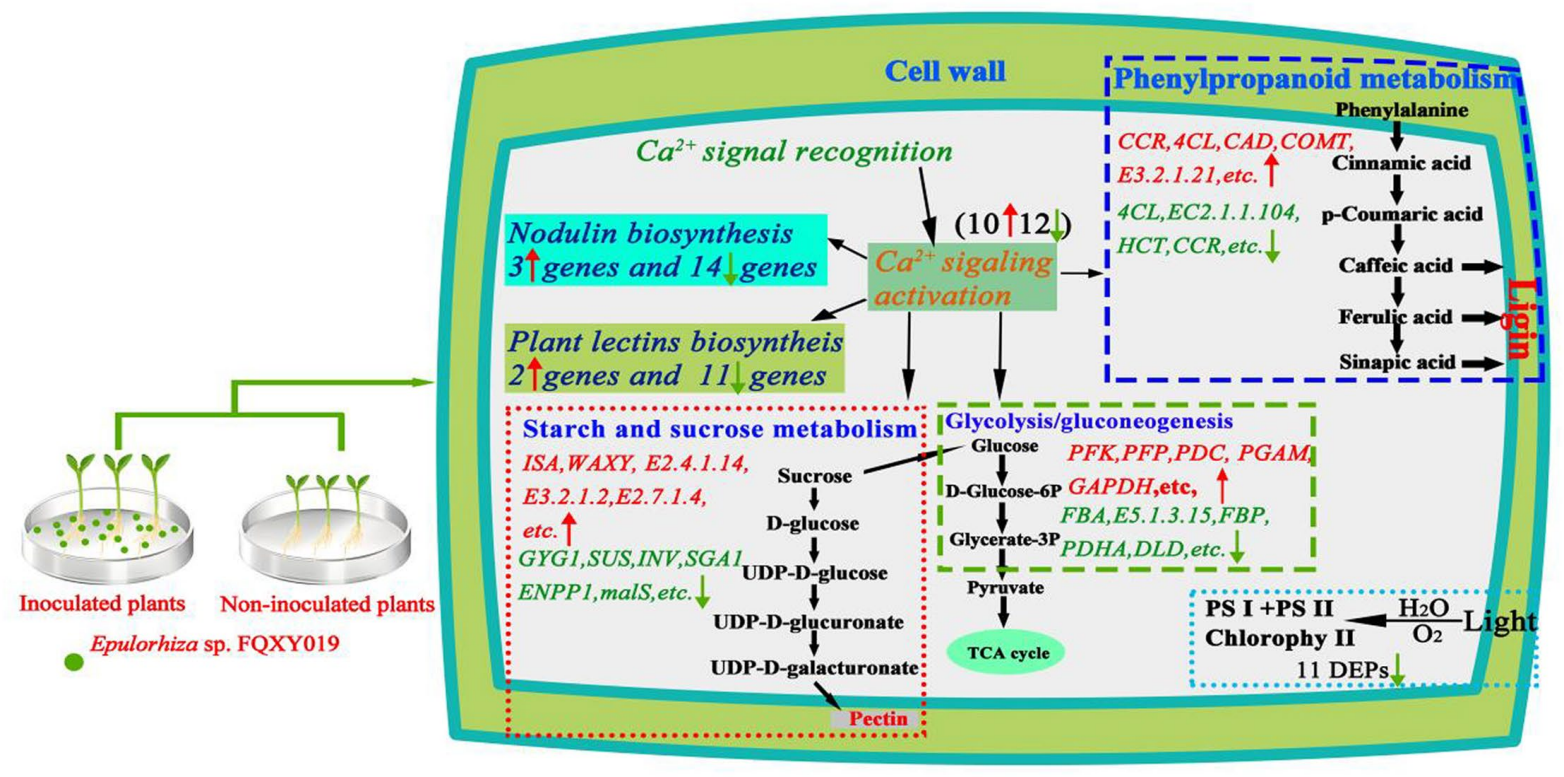
Schematic diagram of the potential mechanisms involved in the effect of *Epulorhiza* sp. FQXY019 symbiosis on the growth of *P. barbigerum*. Red and upward arrows indicate upregulated. Green and downward arrows indicate downregulated.

In this work, a combined transcriptomics, and proteomics analysis was performed to explore the symbiotic interaction of *P. barbigerum* inoculated and uninoculated *Epulorhiza* sp. FQXY019. Lots of DEGs were obtained using transcriptome sequencing during *P. barbigerum* seeds germination. The proteome changes, which were based on TMT labeling-based proteome quantification, also identified a large number of DEPs. Our study demonstrated that symbiosis induced substantial genes and proteins alterations, and regulate growth and development in *P. barbigerum* with FQXY019 (Figure 7). Several studies have proved that the beneficial relationship of carbon exchange in symbionts affects the nutritional balance between fungi and host plants (Li Y. Y. et al., 2021; Zhao et al., 2021). Fungi need to acquire carbohydrates from host plants, thereby regulating nutrient transport to play a dominant role in symbiosis (Vives-Peris et al., 2020). As they lack a mature endosperm, orchid seeds contain only a small amount of carbohydrates as a source of nutrients and need mycorrhizal fungi mycelium to obtain carbohydrates (Zhang et al., 2018; Zhao et al., 2021). However, to adapting to the environment and orchid metabolism, mycorrhizal fungi regulate their carbon and amino acid metabolism and improve their adaptability and survival competitiveness (Zhao et al., 2021).

### 4.1 Transcriptomic analysis

#### 4.1.1 Expression of nodulin-related genes

Nodulation genes are mainly involved in the formation and structural maintenance of nodules, protection of nitrogen fixation activity, and metabolism of nitrogen fixation products in nodule plants, especially in the establishment of the relationship between orchids and mycorrhizal fungi and the development of protocorm symbionts (Zhang et al., 2022). However, these genes not only play a role in nodulation but also in other developmental stages of plants. Several studies reported that nodulation genes and their encoded proteins are activated and actively expressed after arbuscular mycorrhizal fungi infection in plants (Wang et al., 2016; Ghirardo et al., 2020). These genes belong to the phytocyanin family of type I, and the expression products of this family are characterized by binding to type I copper ions and participate in the electron transfer process of the plant–microorganism interaction, plant cell growth, and development (Denance et al., 2014). We identified 17 genes that encoded proteins with a plastocyanin-like domain, which were predicted to be phytocyanin family genes, and one gene that encoded an early nodulation protein through screening the DEGs in CK compared with the FQXY019 treatment group (Supplementary Table S7). In a previous study, the effect of TaCLP1 (a gene encoding plastocyanin protein) on miRNA408 in wheat enhanced the resistance of wheat to stripe rust (Maunoury and Vaucheret, 2011). In addition, nodulin genes were found to be involved in the regulation of nodule formation in Arabidopsis, soybean, and other plants (Svistoonoff et al., 2010; Cheng et al., 2011; Gong et al., 2020). The induced expression of these genes indicated that inoculation could activate the expression of plastocyanin-like domain proteins, enhance the photosynthesis of seedlings, and accelerate seedling growth and development. These genes can also promote the formation of symbiotic nodules, which is beneficial to the uptake of nitrogen by the root system. In conclusion, the study of plant-mycorrhizal fungi interactions involving these genes still needs further study.

#### 4.1.2 Expression of Ca^2+^ signaling-related genes

Ca^2+^ signaling plays a crucial role in regulating the growth and development of plants, such as plant carbon and nitrogen metabolism and ion water transmembrane transport, and the defense response of plants to abiotic environmental stress (Zhang et al., 2022). Ca ions are enriched around the nucleus of root hair cells, which is considered as the earliest cellular response of plants after the establishment of symbiotic relationship (Zhu, 2016). Plant cells have evolved a series of Ca^2+^ responsive receptors, including calmodulin-like protein (CLPs), calcium-binding protein (CBPs), calcium-dependent protein kinases (CDPKs), and calcineurin B-like proteins (CBLs), which can recognize and transmit Ca^2+^ signaling to regulate various cellular activities (De Bang et al., 2021). In this study, numerous genes related to Ca^2+^ signaling were screened, including three DEGs encoding CLPs, eight DEGs encoding CBPs, nine DEGs encoding CDPKs, and one DEG encoding a CBLa (Supplementary Table S8). The protein genes regulated by Ca^2+^ signals are known to be closely related to the formation of arbuscular mycorrhizal symbionts in rice, and the signals generated by these genes can regulate fungal colonization in plant cells (Campos-Soriano et al., 2011). The roots of *Medicago truncatula* activate Ca^2+^ signals and induce the expression of nodule genes to promote root nodule formation after recognizing mycorrhizal factors (such as nodulation factors) released by arbuscular mycorrhizal fungi (Kosuta et al., 2018). In addition, many genes involved in Ca^2+^ signaling have been found in the mycorrhizal symbionts of other orchids. For example, five DEGs encoding CDPKs were reported in the mycorrhizal symbionts of *Epidendrum* (Zhao et al., 2021), and five DEGs encoding CBPs and two DEGs encoding CDPKs were also found in the mycorrhizal symbionts of *Cymbidium hybridum* (Zhao et al., 2014). Therefore, our results indicated that after inoculation with *Epulorhiza* sp. FQXY019, *P. barbigerum* can induce the expression of Ca*^2+^* signaling-related genes, then transfer the signal from the upstream to the downstream gene expression in the root hair area, thus inducing the expression of orchid mycorrhizal symbiotic-related genes and the production of a variety of substances to identify fungi, digest mycelia, and establish an advantageous symbiotic relationship.

#### 4.1.3 Expression of plant lectin-related genes

Plant lectins are a class of nonenzymatic proteins found in legumes that can agglutinate cells and precipitate monosaccharide or polysaccharide complexes (Kosuta et al., 2018; Ahmad et al., 2022b). Plant lectins play a crucial role in many signaling processes, for instance, signal transduction, plant defense, and immune response. Recent studies have reported that the specific recognition of plant symbiosis may be mediated by lectins, which can specifically bind carbohydrate complexes owing to their specific ability to bind monosaccharides and carbohydrate complexes, such as with mannitol-specific lectins (Houston et al., 2016; Andrade et al., 2019; Valadares et al., 2020). In this study, 13 DEGs were found to encode lectin-related proteins in the symbiotic mechanism of *P. barbigerum* between the FQXY019 treatment and CK group, six of which were downregulated DEGs that encoded mannose-specific lectin proteins (Supplementary Table S9). Our results were similar to previous studies. For example, significantly upregulated expression of mannose-specific lectin genes was found in several studies of orchid–fungi symbiotic relationships, such as between a *Cymbidium* hybrid and six different fungal strains (Reinhold-Hurek et al., 2015), *Platanthera sikkimensis, T. calospora* and *Epidendrum secundum* (Pujasatria et al., 2020). In addition, a mannose-specific lectin was reported to be involved in the establishment of the interaction with microorganisms during *Gastrodia elata* seed germination (Zeng et al., 2017). The induced expression of these genes indicates that they are one of the key factors for the establishment of symbiotic relationship between orchids and mycorrhizal fungi. Mycorrhizal fungi have faster growth and development than orchid plants and can quickly invade plant cells to establish a symbiotic relationship with plants (Dong et al., 2008; Xu et al., 2017). During this process, numerous plant lectin-related genes were induced and expressed, which played an important role in regulating and maintaining the interaction between orchids and mycorrhizal fungi.

### 4.2 Proteomic analysis

#### 4.2.1 Deps related to photosynthesis and energy-associated metabolism

Although most orchids can obtain carbon via photosynthesis, they still need fungi to provide nutrients. Zhang et al. (2020) found that *Mycena* increased the expression of photosynthesis-related proteins, such as antenna proteins, in *Dendrobium officinale*, thereby enhancing the photosynthetic efficiency of *D. officinale*. In a study on the symbiotic relationship between *Oncidium sphacelatum* and *Ceratobasidium*, the expression of photosynthesis-related proteins in the green protocorm of *O. sphacelatum* was found to be upregulated, including that of ribulose diphosphate carboxylase and chlorophyl a-apolipoprotein A1 of photosystem I, which affected their own photosynthesis. However, in this study, we observed that the expression of 13 DEPs related to photosynthesis was greatly downregulated in the CK in comparison with that in the FQXY019 treatment groups (Supplementary Table S11). In addition, eight DEPs related to photosynthesis antenna proteins were markedly downregulated in the CK in comparison with that in the FQXY019 treatment groups (Supplementary Table S12). These resulted indicate that the photosynthesis of *P. barbigerum* was gradually changed during the symbiotic development with *Epulorhiza* sp. FQXY019. Thus, this enables the energy requirement of seeding germination to be met with adjustment of the seed germination rate to quickly enable growth of seedlings of *P. barbigerum*.

During plant seed germination, starch in the endosperm is gradually degraded as the main source of ATP, which provides a precursor for the biosynthetic and metabolic reactions in embryos (Zhao et al., 2021). Nevertheless, orchid seeds are very small and nutrient storage is limited, and seed germination and subsequent developments of orchids in nature mainly depend on the colonization of mycorrhizal fungi (Li Y. Y et al., 2021). After the mycorrhizal fungi colonize the orchid seeds, the mycelium passes through the seed coat and produces amylase to hydrolyze starch in the embryo, which promotes the growth of the embryo protoplasm and absorbs nutrients from the surrounding environment to accelerate the further germination and growth of seeds (Pujasatria et al., 2020). In this study, transcriptomics showed that two pathways (starch and sucrose metabolism, and glycolysis/gluconeogenesis) related to energy metabolism were significantly enriched, involving a total of 72 DEGs, all of which were upregulated in the CK in comparison with the FQXY019 treatment groups (Supplementary Tables S13, S14). Interestingly, proteomics also showed that two pathways (pentose and glucuronate interconversions, and glycolysis/gluconeogenesis) related to energy metabolism were enriched and included a total of 31 DEPs, of which 20 DEPs were upregulated and 11 DEPs were downregulated in the CK in comparison with that in the FQXY019 treatment groups (Supplementary Tables S15, S16). These results demonstrated that the energy metabolism-related pathways in seeds of *P. barbigerum* rapidly changed after the seeds were infected by *Epulorhiza* sp. FQXY019, and the seeds germinated and formed seedlings. The energy metabolism pathways in seedlings also changed during the symbiotic development of seedlings and mycelium to meet the energy required for seedling growth, which is consistent with results from previous studies (Fochi et al., 2017).

#### 4.2.2 Deps related to fatty acid metabolism

Orchid seeds are among the smallest seeds in nature, and they are naturally rich in fatty acids (Chen et al., 2018). Fatty acids can generate intermediate products of sugar metabolism via glyoxylate and dicarboxylate metabolism and other metabolic processes and then convert them into carbohydrates via gluconeogenesis, which can provide sufficient energy for seed germination by glycolysis and pentose phosphate pathway (Ghirardo et al., 2020). Therefore, in this study, the DEGs and DEPs involved in the fatty acid metabolism in *P. barbigerum* were analyzed. The transcriptomics analysis showed that a total of 11 upregulated DEGs involved in fatty acid degradation, and 16 and 8 of the downregulated DEGs involved in fatty acid elongation and biosynthesis of unsaturated fatty acids, respectively, in the CK in comparison with that the FQXY019 treatment groups (Supplementary Table S17). A total of 13 DEPs (seven upregulated and six downregulated DEPs) were involved in fatty acid biosynthesis and metabolism in proteomics in the CK in comparison to the FQXY019 treatment groups, respectively (Supplementary Table S18). In addition, we found that two DEPs (one upregulated and one downregulated DEP) were related to linoleic acid metabolism in the CK in comparison with that in the FQXY019 treatment groups (Supplementary Table S18). These results indicate that inoculation with *Epulorhiza* sp. FQXY019 can affect the synthesis, decomposition, and metabolism of fatty acids in seeds of *P. barbigerum* by invading into seeds, so that the fatty acids stored in embryos can be more efficiently used to pass through the early stage of germination. Chen et al. (2022) found that the colonization by fungi could change the metabolic pattern and improve the nutrient utilization efficiency of *D. officinale*, especially when lipid metabolism was activated. In addition, genes encoding key enzymes involved in fatty acid metabolism, such as acetyl-CoA binding protein and β-oxidation polyfunctional protein genes, were significantly upregulated. Overall, our results indicated that the *Epulorhiza* sp. FQXY019 can induce the expression of genes and proteins related to fatty acid metabolism in *P. barbigerum*, and that the carbohydrates produced by this process can provide energy resources for seed germination and seedling growth.

## 5 Conclusion

This study demonstrated that inoculation of *Epulorhiza* sp. FQXY019 boosted and changed the transcriptomic and proteomic profile of *P. barbigerum* seed germination. Based on transcriptional evidence, numerous DEGs were identified in the CK and FQXY019 treatment groups, which are mainly associated with sugar, fatty acid, and amino acid metabolic pathway The expression levels of candidate DEGs related to nodulin, Ca^2+^ signaling, and plant lectins were significantly affected in *P. barbigerum* with FQXY019 treatment. Additionally, a total of 537 DEPs were identified in proteomic analysis and were involved in carbon metabolism, amino acid biosynthesis, and fatty acid metabolism. We identified several candidate DEPs related to photosynthesis, energy metabolism, and fatty acid metabolic pathways. Collectively, the identified genes and proteins will provide insight into the specific mechanism underlying the fungal inoculation of *Paphiopedilum* orchid seeds and their subsequent germination, and will act as a constructive reference resource to improve the genetic improvement of *Paphiopedilum* orchids.

## Data availability statement

The datasets presented in this study can be found in online repositories. The names of the repository/repositories and accession number(s) can be found in the article/Supplementary material.

## Author contributions

FT: Writing – original draft, Software, Investigation, Data curation. JW: Writing – original draft, Software, Methodology, Investigation, Data curation. FD: Writing – review & editing. LW: Writing – review & editing. YY: Writing – review & editing. XB: Writing – review & editing. CT: Writing – review & editing. XL: Writing – review & editing.

## Glossary

AGC: Automatic gain control
BP: Biological process
CBL: Calcineurin B-like
CBP: Calcium-binding protein
CC: Cellular component
CDPK: Calcium-dependent protein kinases
CLP: Calmodulin-like protein
DEG: Differentially expressed genes
DEP: Differentially expressed proteins
FA: Formic acid
FDR: False discovery rates
GO: Gene Ontology
KEGG: Kyoto Encyclopedia of Genes and Genomes
MF: Molecular function
MS: Mass spectrometry
NCBI: National Centre for Biotechnology Information
PCA: Principal component analysis
PDA: Potato dextrose agar
TMT: Tandem mass tag
