# Supplementary material for: Comparative transcriptomics and proteomics analysis of the symbiotic germination of Paphiopedilum barbigerum with Epulorhiza sp. FQXY019
Source: Front Microbiol. 2024 Mar 18;15:1358137. doi: 10.3389/fmicb.2024.1358137 (PMC10982344; doi:10.3389/fmicb.2024.1358137)
Supplement: Supplementary file 1 [file Data_Sheet_1.ZIP › Figure S1-S12.docx]

| 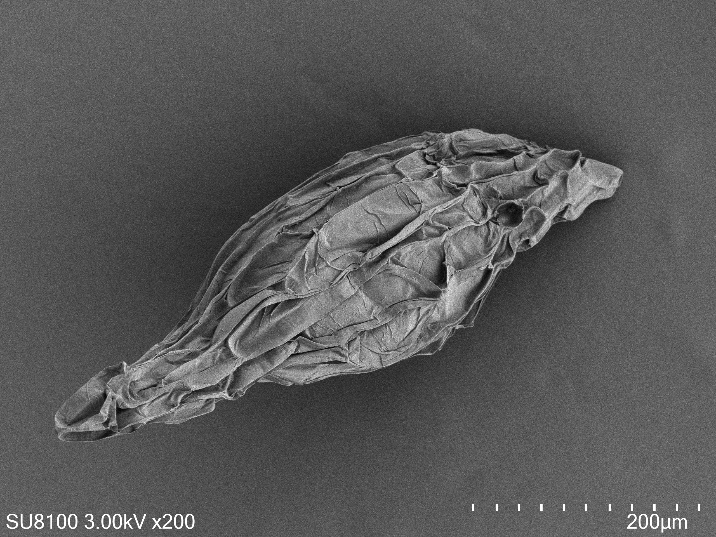  c  a | 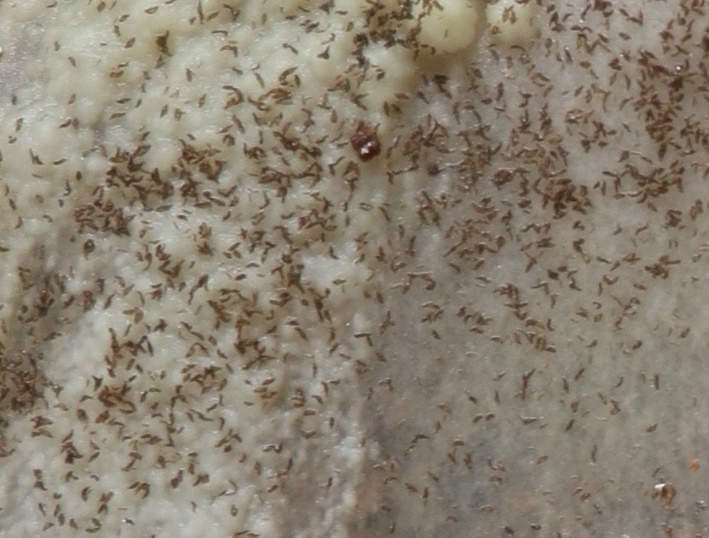  d  b |
| --- | --- |
| 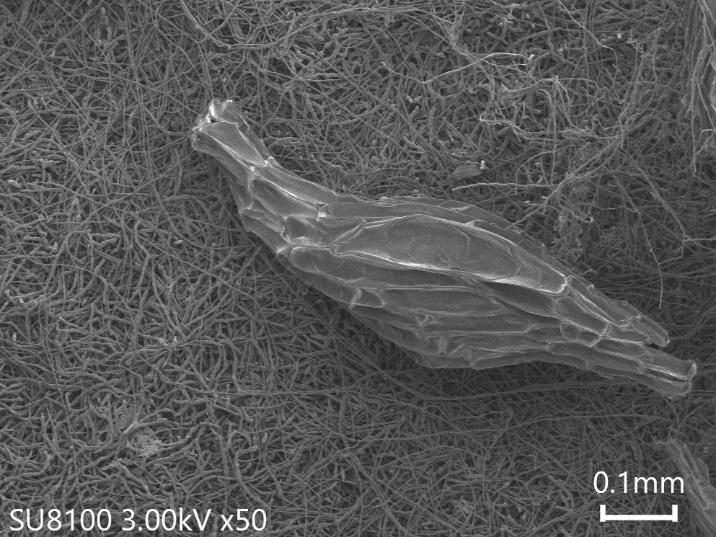  **Mass clusters of monilioid cell chains** | 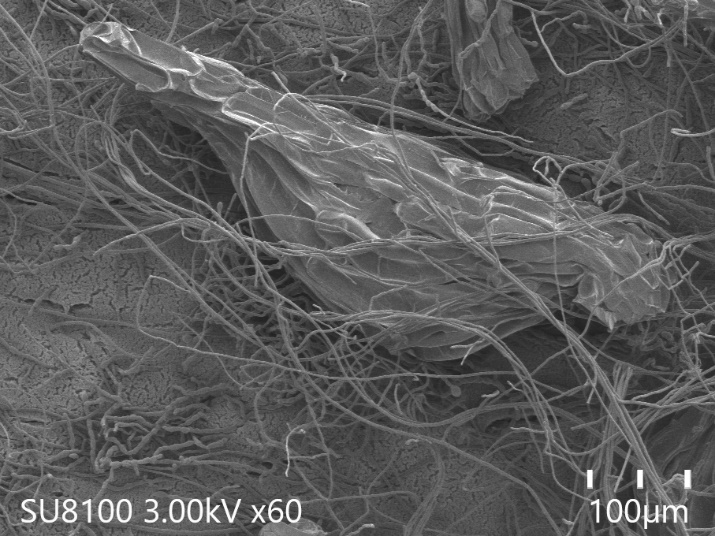  **Hyphae** |
| 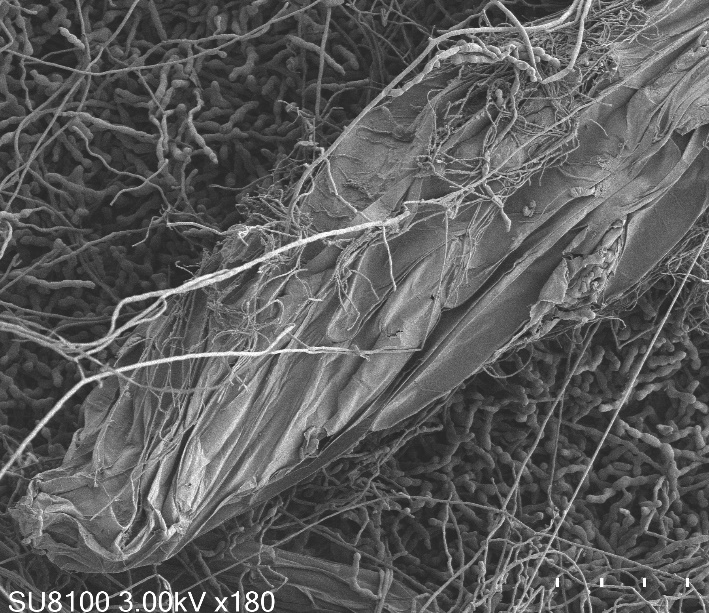  **Monilioid cells broke through the seed coat**  **Monilioid cells** **chain** | 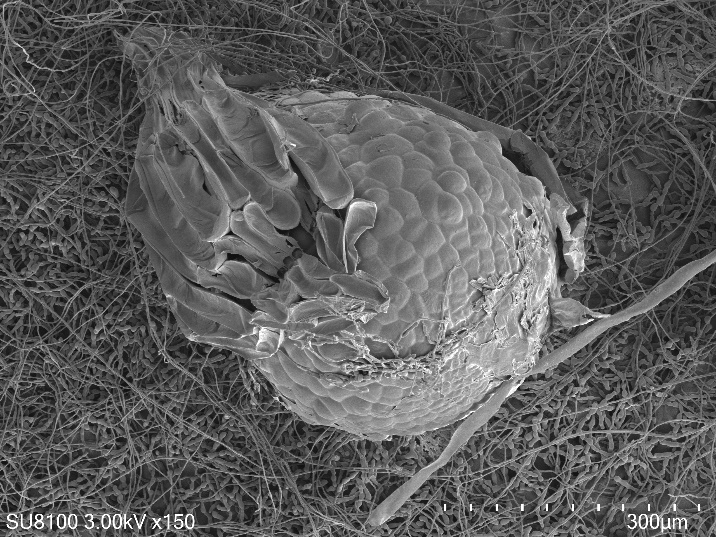  f  **Shoot apical meristem** |
| 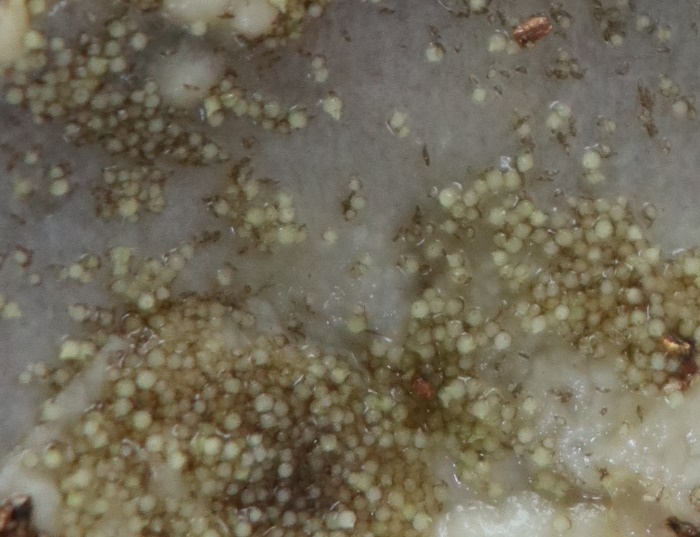  **Leaf primordia**  i  g | 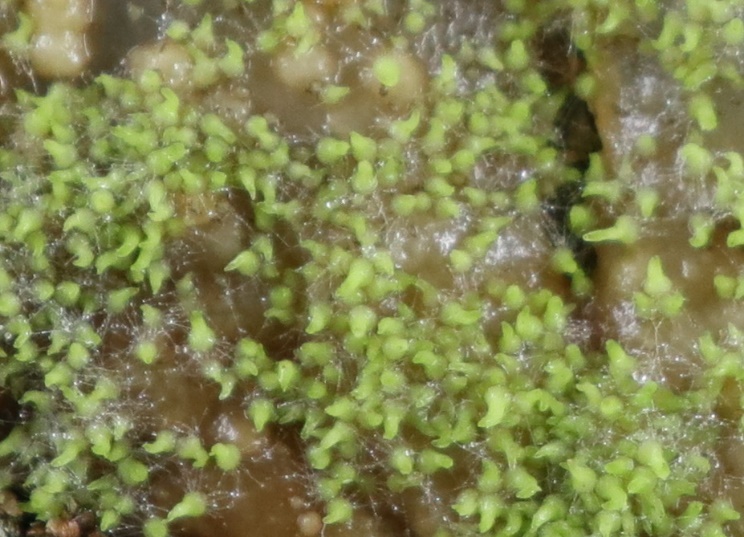  **Cilium rhizoids**  **Emergence of leaf**  j  h |
| 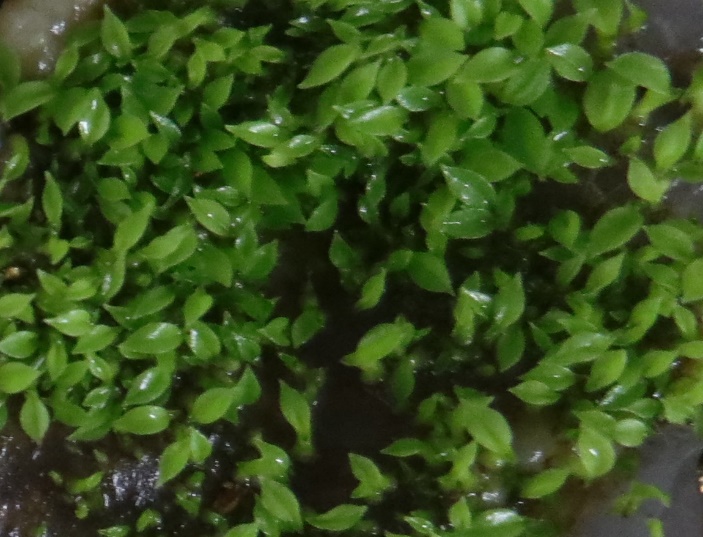  **Elongation of leaf to form seedling** | 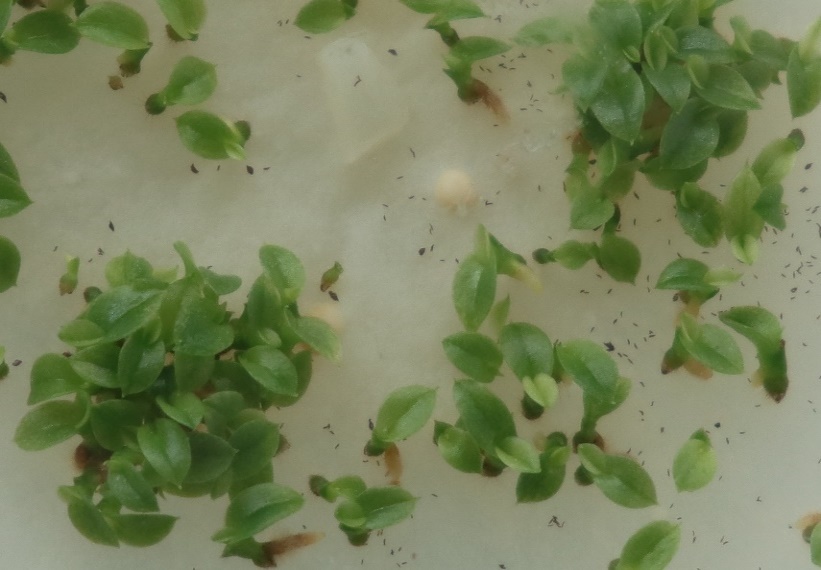  **Rooting** |

e

**Figure S1.** SEM and camera images of symbiotic seed germination and seedling developmental stages of *Paphiopedilum* *barbigerum* inoculated with *Epulorhiza* sp. FQXY019.Mature seed of *P. barbigerum* (Fig. S1 a). Seeds are inoculated onto mycelium (Fig. S1 b,c). Hyphae and monilioid cells wrapped around the seed coat(arrow) (Fig. S1 d). Hyphae and monilioid cells broke through the seed coat (arrow) (Fig. S1 e). Embryo developed into protocorm, and the embryo polarity was established by development of the protomeristem at the apex, with shoot apical meristem appeared (arrow) (Fig. S1 f). .Emerged cilium rhizoids and leaf primordia(Fig. S1 g). Differentiate into the first leaf (arrow) (Fig. S1 h). Elongation of the leaf and rooting to form a seedling(Fig. S1 i,j).


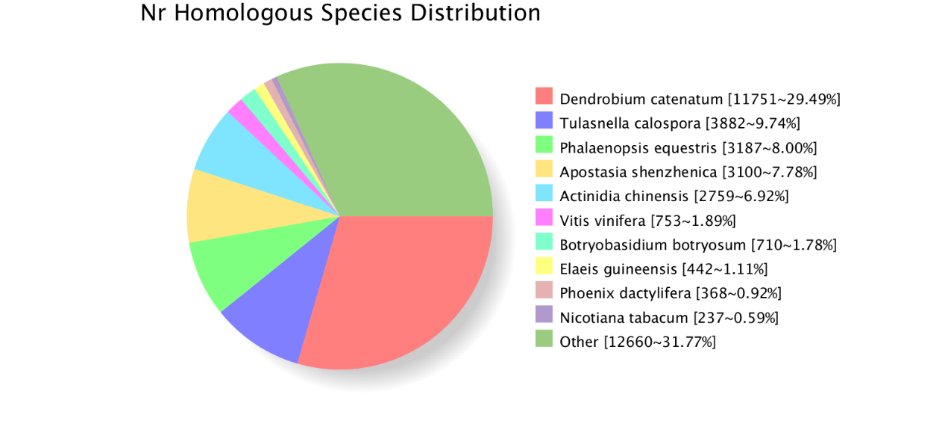


**Fig. S2** The homologous species distribution.


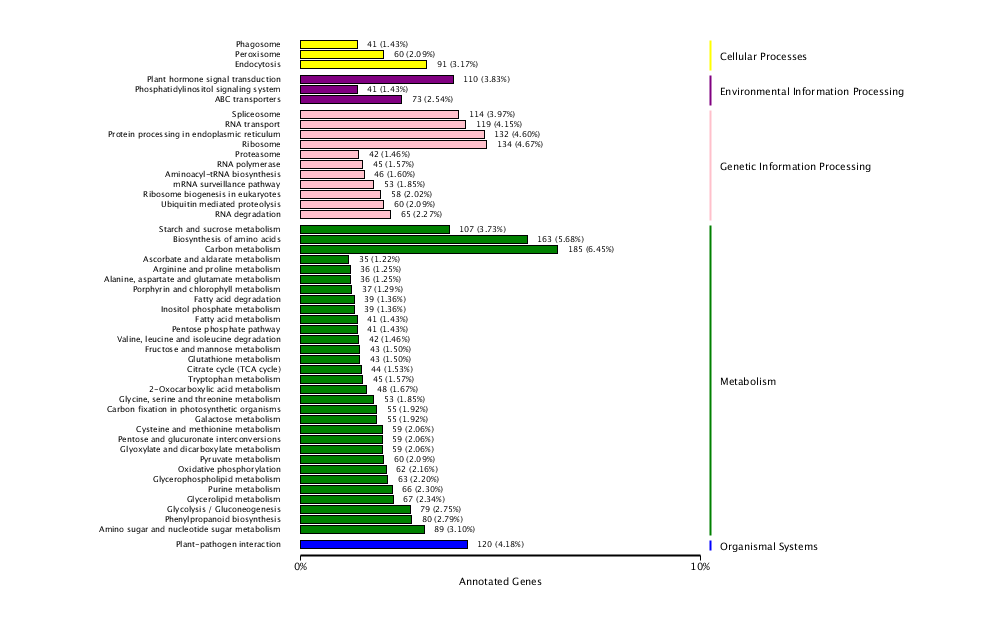


**Fig. S3** KEGG annotation analysis of DEGs.


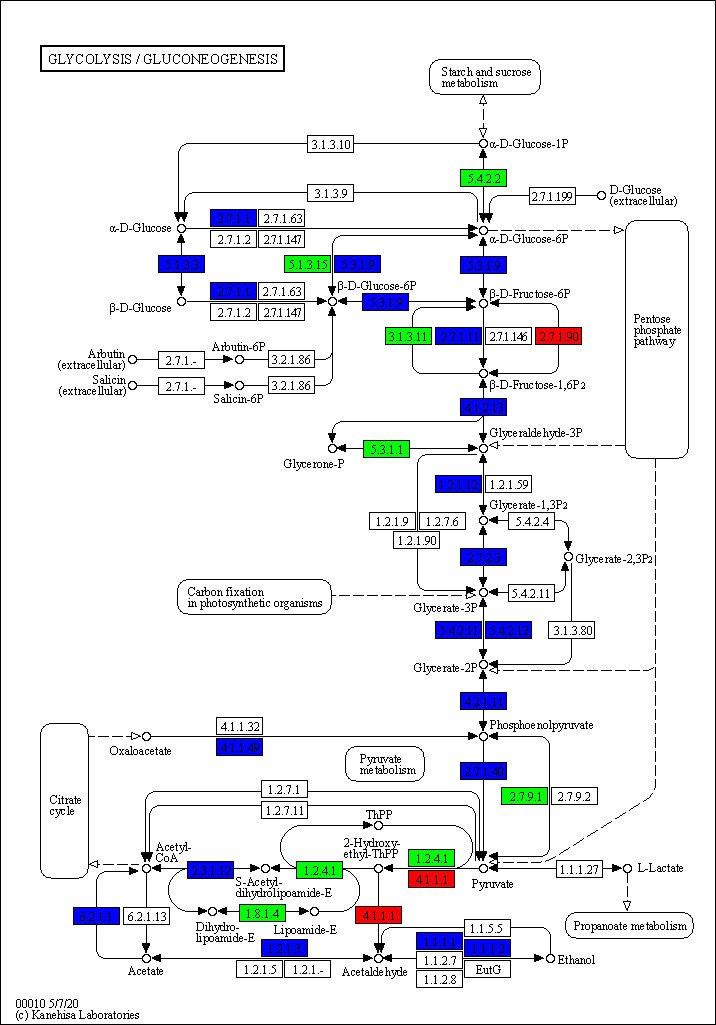


**Fig. S4** The KEGG pathway of glycolysis/gluconeogenesis

Note: red and green texts in the solid line box mean upregulated and downregulated enzymes corresponding to the transcript, respectively, and the blue texts in the solid line box mean both upregulated and downregulated enzymes.


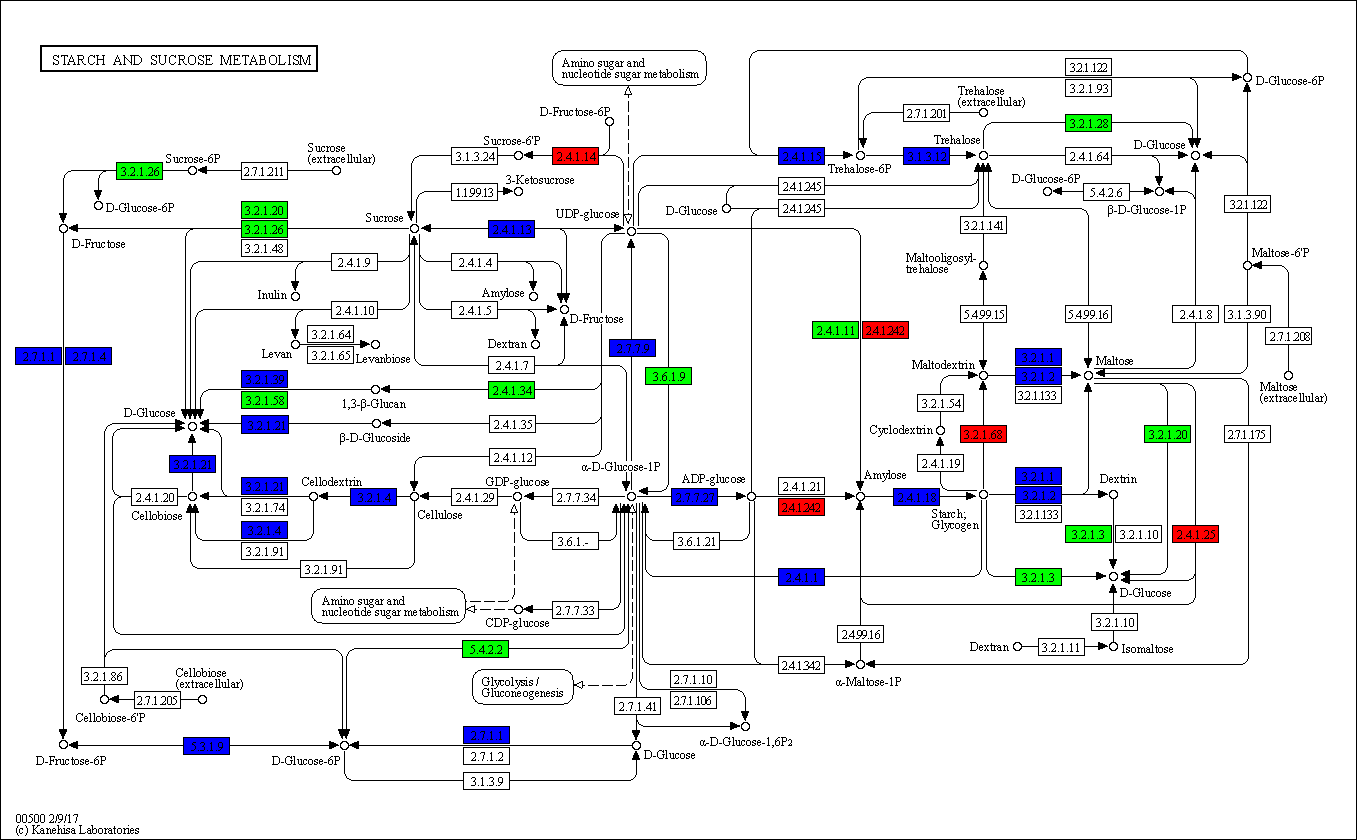


**Fig. S5** The KEGG pathway of starch and sucrose metabolism

Note: red and green texts in the solid line box mean upregulated and downregulated enzymes corresponding to the transcript, respectively, and the blue texts in the solid line box mean both upregulated and downregulated enzymes.


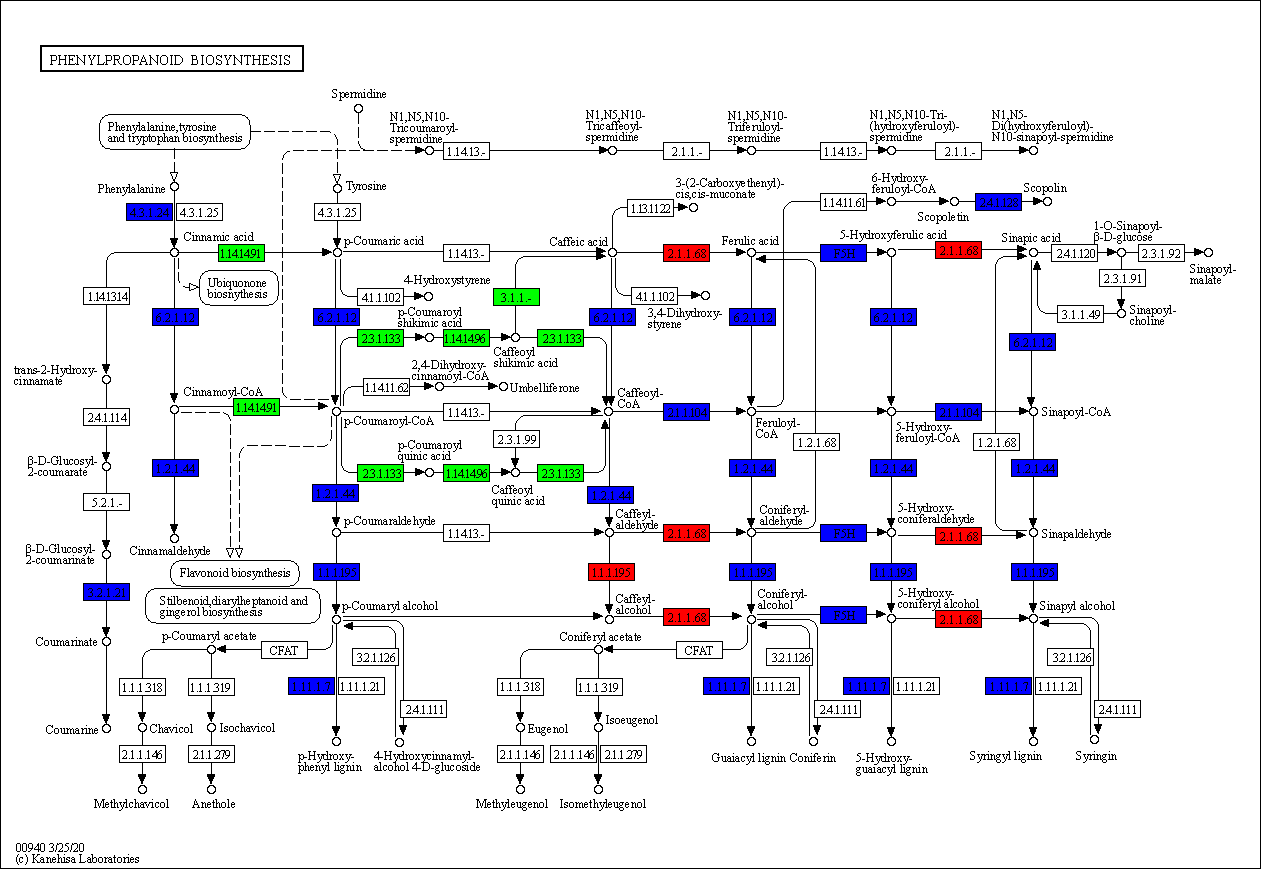


**Fig. S6** The KEGG pathway of phenylpropanoid biosynthesis

Note: red and green texts in the solid line box mean upregulated and downregulated enzymes corresponding to the transcript, respectively, and the blue texts in the solid line box mean both upregulated and downregulated enzymes.


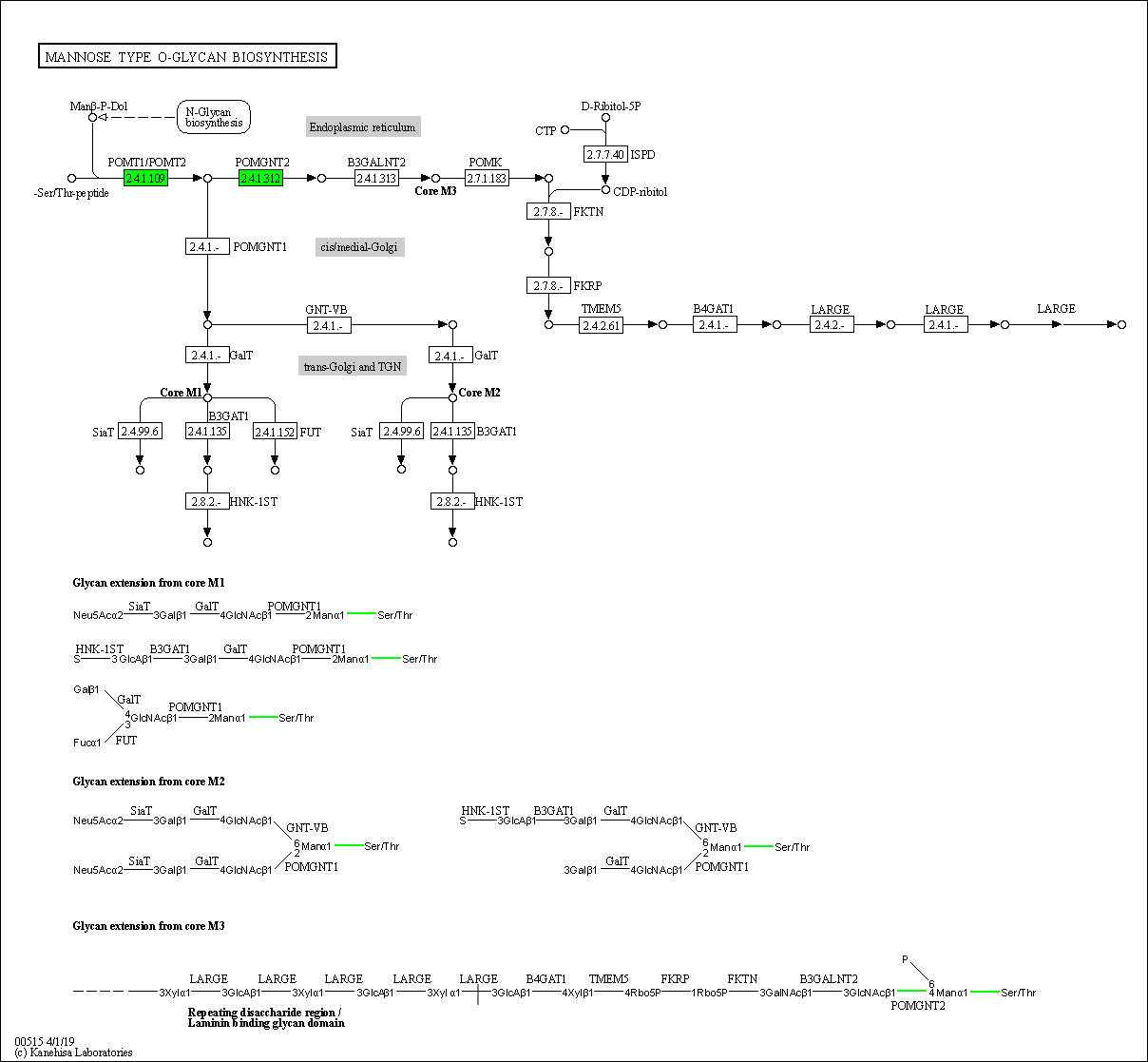


**Fig. S7** The KEGG pathway of mannose type O-glycan biosynthesis

Note: green texts in the solid line box mean downregulated enzymes corresponding to the transcript.


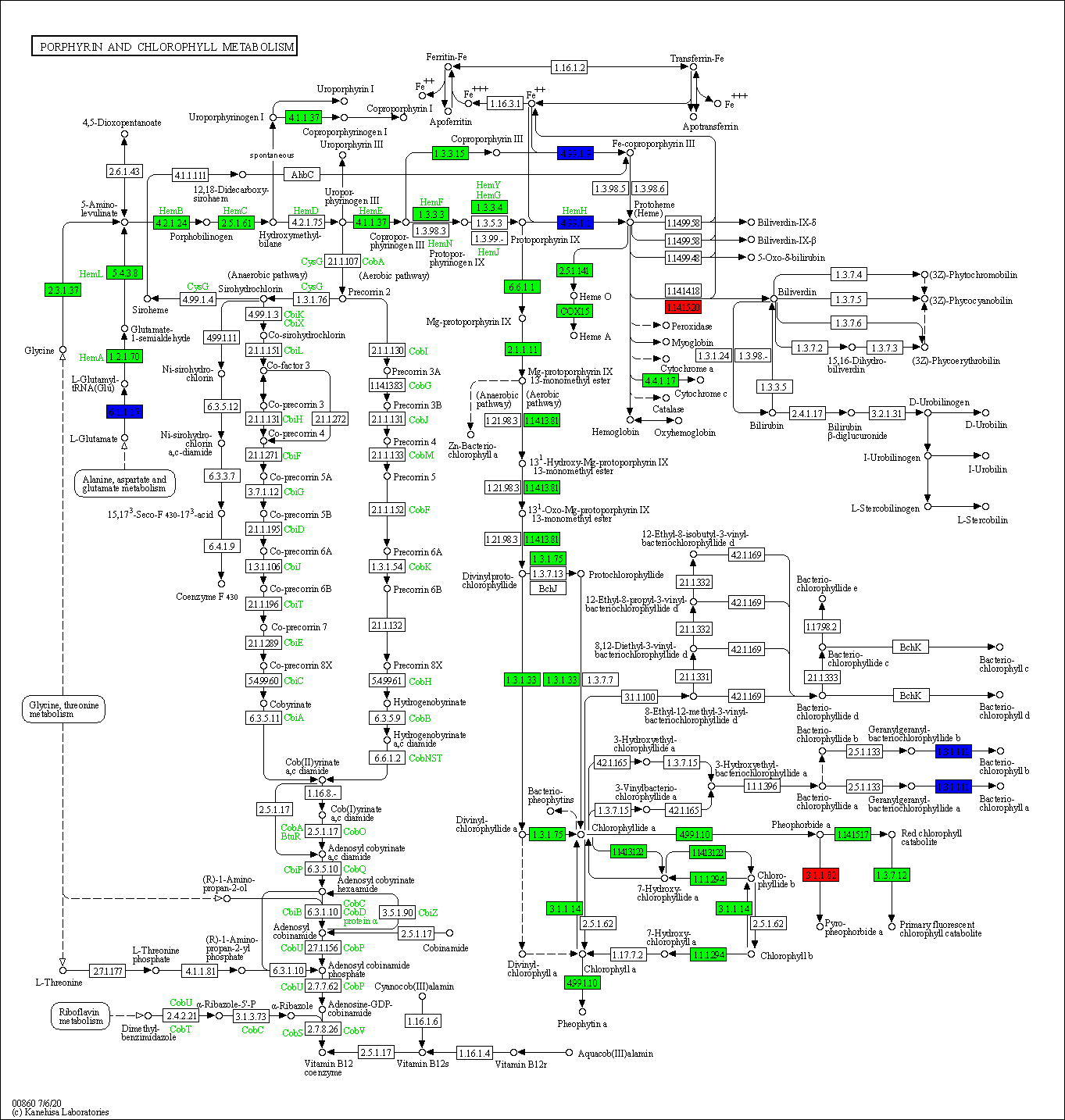


**Fig. S8** The KEGG pathway of porphyrin and chlorophyl II metabolism


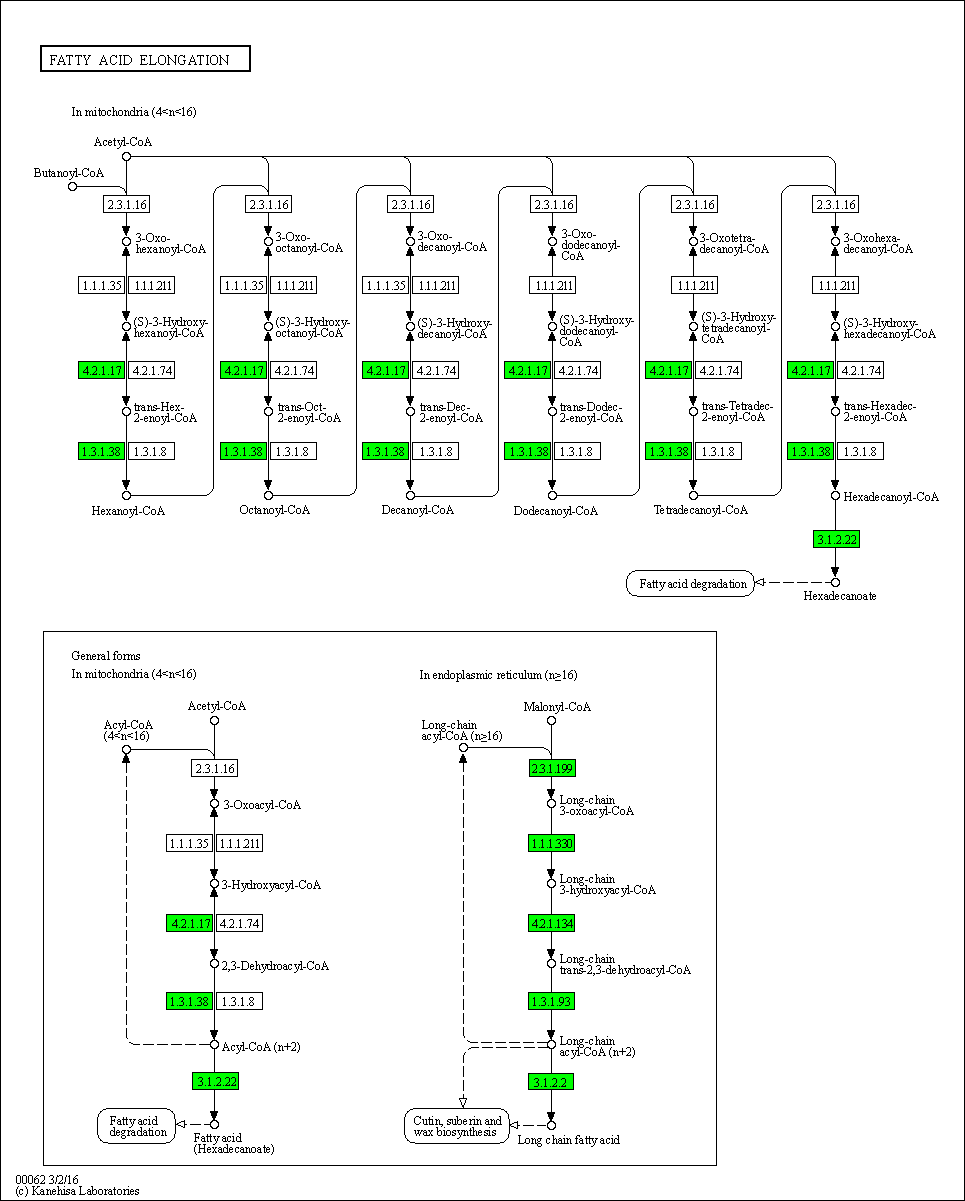


**Fig. S9** The KEGG pathway of fatty acid elongation


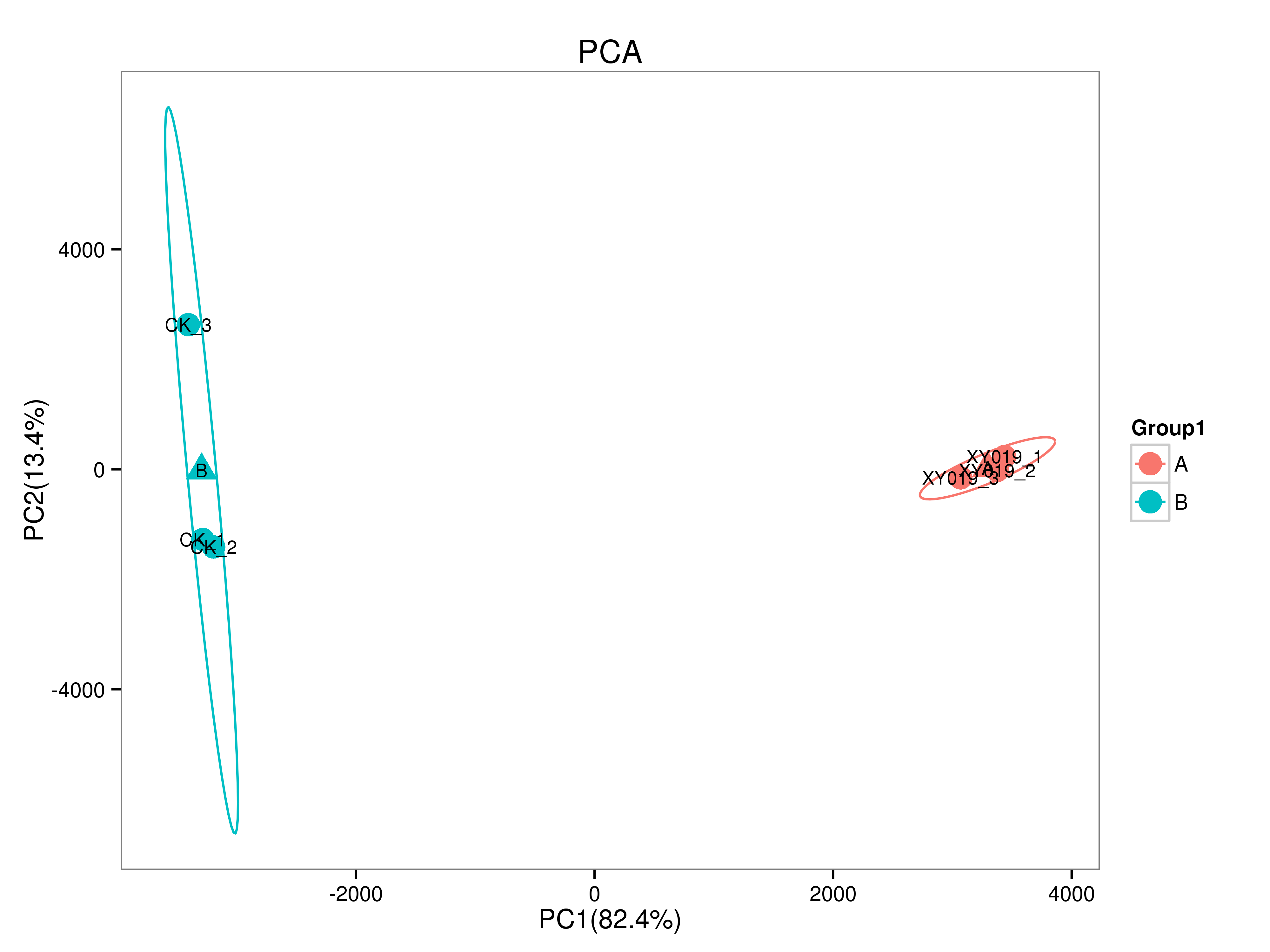


**Fig. S10** Principal component analysis (PCA) of identified proteins in control and inoculation groups.


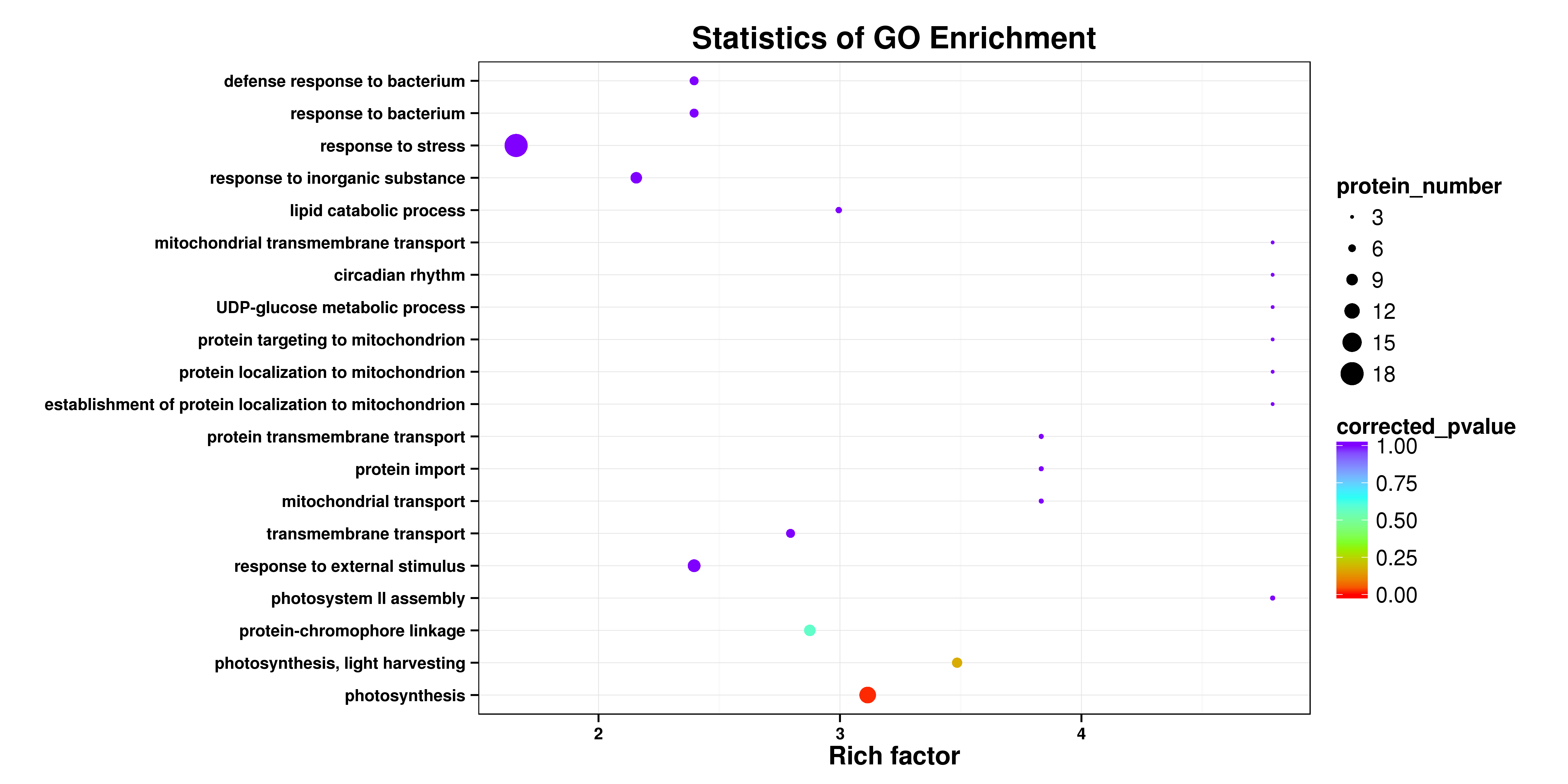

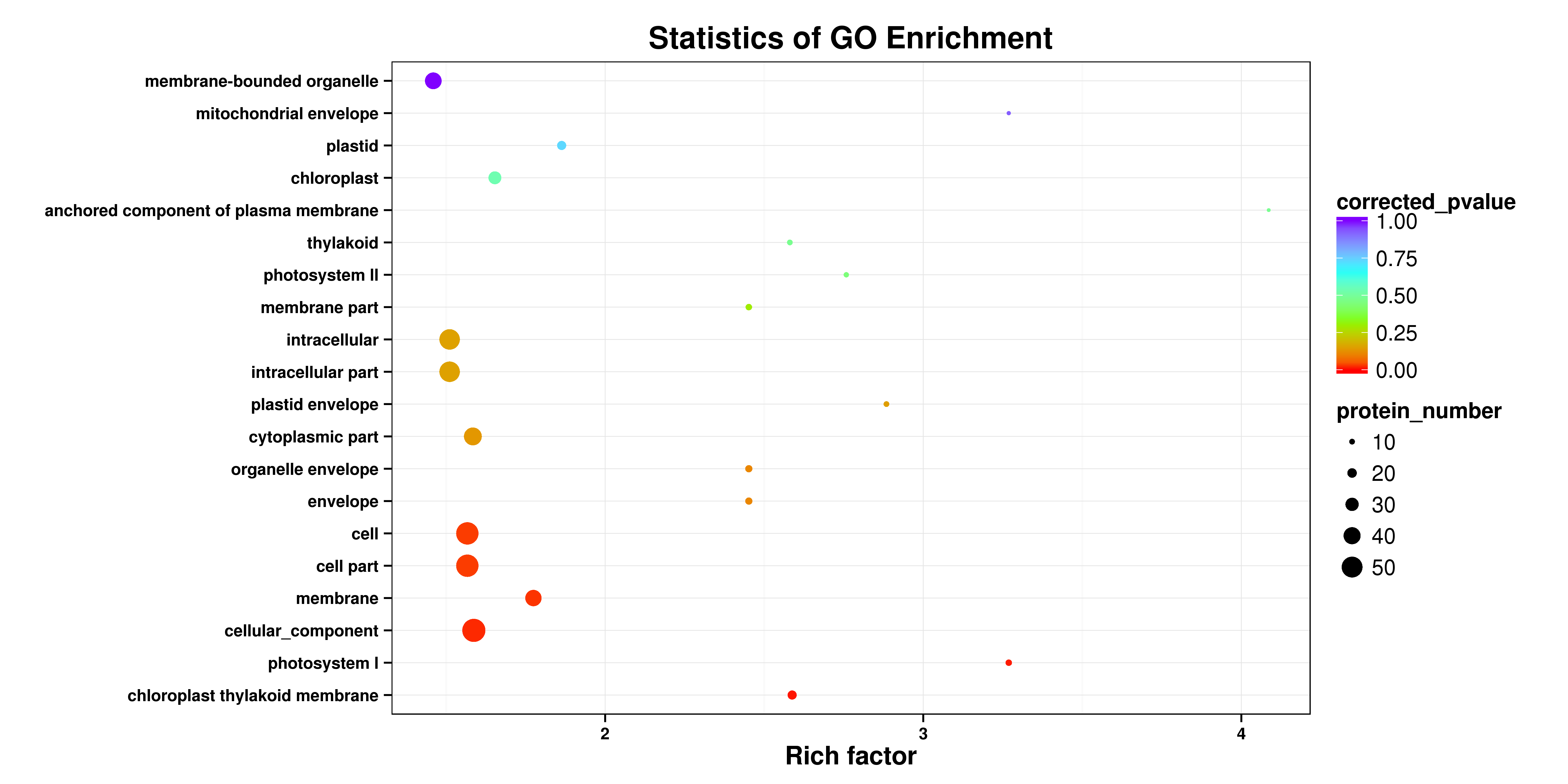


A

B


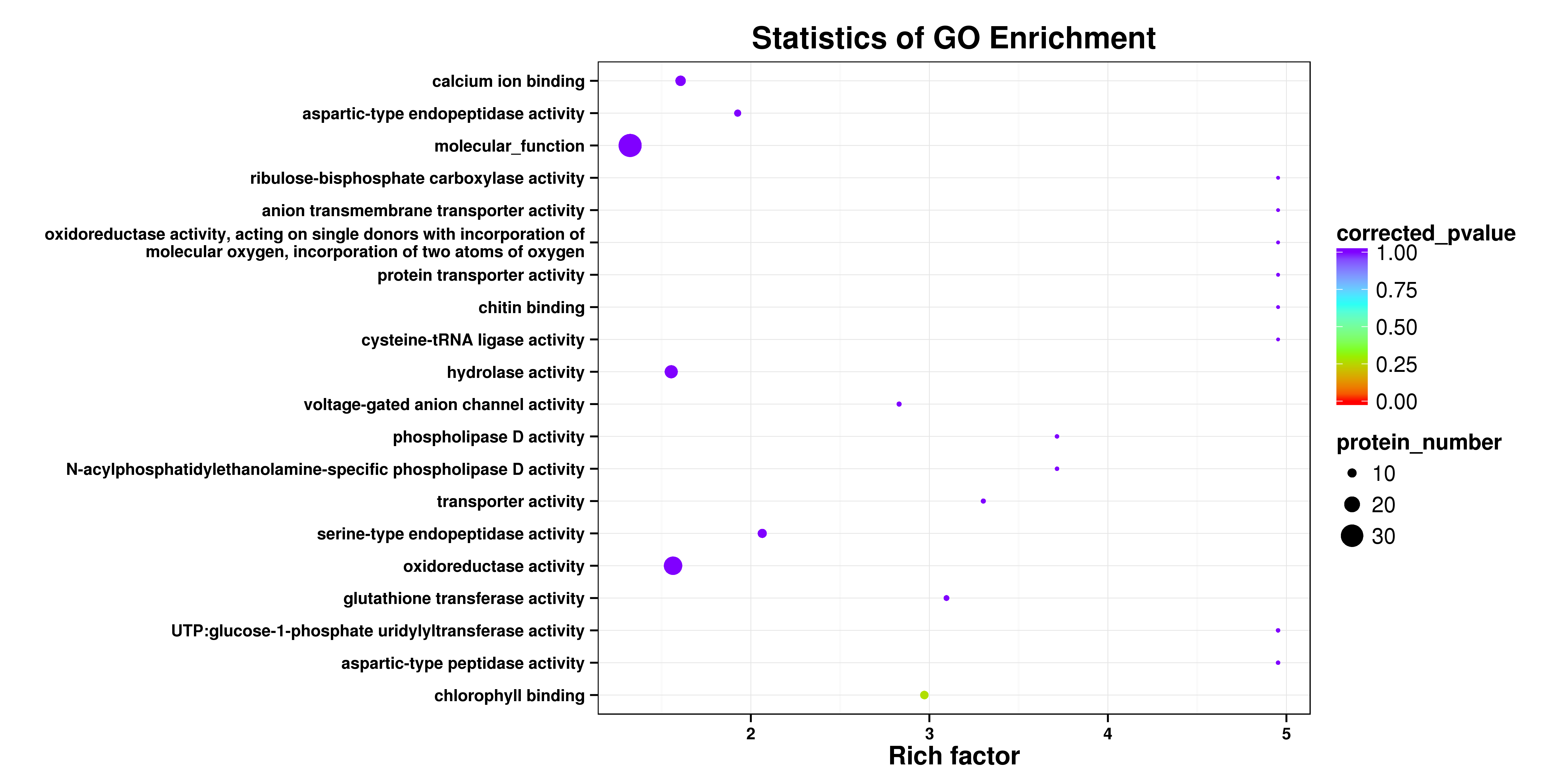


C

**Fig. S11** GO enrichment analysis of DEPs. **(A)** Biological process, **(B)** Cellular component and **(C)** Molecular function.

Note: The X-axis was the rich factor, which was the ratio of the DEP number to the total proteins number in a certain pathway; the Y-axis represents the name of the pathway. The bubbles size represents the number of DEPs involved. The bubbles color indicates the enrichment degree of pathway.


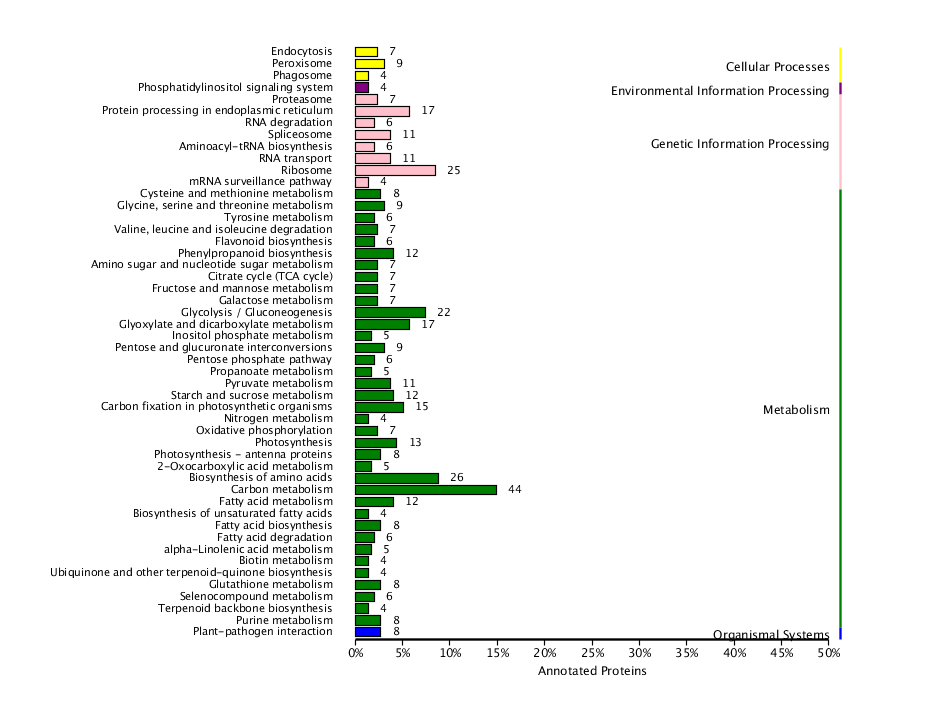


**Fig. S12** KEGG annotation analysis of DEPs.
